# Usable comprehensive-factor authentication for a secure time attendance system

**DOI:** 10.7717/peerj-cs.678

**Published:** 2021-08-16

**Authors:** Chalee Vorakulpipat, Sasakorn Pichetjamroen, Ekkachan Rattanalerdnusorn

**Affiliations:** Information Security Research Team, National Electronics and Computer Technology Center, Pathumthani, Thailand

**Keywords:** Information security, Multi-factor authentication, Access control, Usability, Time attendance, Mobile device

## Abstract

In information security, it is widely accepted that the more authentication factors are used, the higher the security level. However, more factors cannot guarantee usability in real usage because human and other non-technical factors are involved. This paper proposes the use of all possible authentication factors, called comprehensive-factor authentication, which can maintain the required security level and usability in real-world implementation. A case study of an implementation of a secure time attendance system that applies this approach is presented. The contribution of this paper is therefore to provide a security scheme seamlessly integrating all classical authentication factors plus a location factor into one single system in a real environment with a security and usability focus. Usability factors emerging from the study are related to a seamless process including the least number of actions required, the lowest amount of time taken, health safety during the pandemic, and data privacy compliance.

## Introduction

Identifying a user with a multi-factor authentication scheme is widely accepted to ensure that credentials are not stolen by an unauthorized person or robot. Current multi-factor authentication consists of three classical categories: *something you know* (*e.g*., password), *something you have* (*e.g*., key, Radio-Frequency Identification or RFID, smartphone One-Time Password or OTP), and *something you are* (biometric data, *e.g*., fingerprint, facial data, iris). Recently, *somewhere you are* or location has been included as an additional factor to detect or track the user’s location from a tracking device (*e.g*., Global Positioning System-GPS, WiFi locator, RFID reader location, etc.), although it is not as widely accepted as the other three factors (Boonkrong, 2021a). Nevertheless, *somewhere you are* can at the least be used as a supplement to other factors. It is understood that the more factors are used, the higher the level of security. A number of factors, in particular biometric data, have been adopted to ensure security in high-tech systems (Shalaby et al., 2021). Too much security cannot confirm usability, however, (De Paula et al., 2005) because many issues such as speed, cost, and privacy inhibit real implementation. Users appreciate not only security but also functionality, especially ease of use (Vorakulpipat, Pichetjamroen & Polprasert, 2021). Many systems deploy multiple factors to ensure security, but users encounter problems with system performance and find it time consuming.

During the COVID-19 pandemic, there have been more restrictions on access control because health safety has been taken into account (Vorakulpipat, Pichetjamroen & Polprasert, 2021). An access control system is required to follow health safety norms, such as social distancing, contactless solutions, or using users’ own devices. In addition, data privacy in personal information is another issue due to compliance with laws (Stallings, 2020) including the General Data Protection Regulation (GDPR) in the European Union (EU), the California Consumer Privacy Act (CCPA), and the Personal Data Protection Act (PDPA) in Thailand, all of which may hinder the use of biometric data. Therefore, using multiple-factor access control or strong security mechanisms with these restrictions is difficult to make practical (Stevens et al., 2020). Impractical security has been mentioned in some studies (Ye & Qian, 2017; Yan, Deng & Varadharajan, 2017), but most of the problems explored have been related to system performance (such as advanced cryptography) rather than user acceptance. In this paper, we propose a usable scheme to authenticate users by using all three classical factors plus *somewhere you are*. The proposed mechanism is deployed as a time attendance application used in a technology park.

The contribution of this paper is to provide a novel security mechanism that emphasizes the usability of an integration of all authentication factors in a real environment. The paper does not aim to focus on the accuracy of each authentication factor as is found in existing literature and does not propose a new single authentication factor.

The paper first presents a literature review of related works focusing on multi-factor authentication, an attendance system with a security scheme, and usability in security systems. This is followed by our proposed comprehensive-factor time attendance system demonstrating necessary processes and system design. The results section and discussion section are then presented. Conclusions including future recommendations are provided in the final section.

## Related works

Multi-factor authentication, which means the use of at least two different factors, aims to increase the level of information security, privacy, and trust in particular in advanced environments, such as mobile devices and clouds (Anakath, Rajakumar & Ambika, 2019). Factors influencing the adoption of multi-factor authentication have been studied. However, these factors do not only include technical concerns like security, privacy, and trust management, but also perception issues such as ease of use, efficiency, reliability, and human trust (Mohsin et al., 2017). Importantly, in smart hyper-connected devices and wearable devices, a user needs seamless and user-friendly authentication procedures; thus, this is a challenge where system developers must consider the pros and cons in terms of technical and human aspects (Ometov et al., 2018; Ometov et al., 2019). Compliance with privacy regulations and concerns is also considered when implementing information systems in critical systems involving personal data (Boonkrong, 2021b), such as online banking (Sinigaglia et al., 2020) and health informatics (Vorakulpipat et al., 2019).

It has been confirmed that multi-factor authentication is more widely used in mobile environments and smart contexts. The use of biometric methods and smartphone one-time password (OTP) is commonly suggested in mobile apps (Maciej & Kurkowski, 2019). A number of research studies have attempted to introduce new authentication methods in multi-factor authentication. A CCTV and smartphone-based multi-factor authentication framework using face detection/recognition and unique hardware identification has been proposed (Kwon, Sharma & Park, 2019) as well as a combination of the use of mobile device and fingerprint (Mohammed & Yassin, 2019) methods. In an online examination regarding *something you know*, the scheme involves people sharing information prior to the exam date and answering questions relating to the shared information just before the exam (Ullah, Xiao & Barker, 2018). In Automatic Teller Machines (ATM), adopting all factors is possible (Abiew, Jnr & Banning, 2020), whereas an uncommon biometric method, iris recognition, has been proposed to practically authenticate ATM users (Akinola Kayode et al., 2019). Machine learning is one important technology that is used in multi-factor authentication to help detect fraud in mobile devices, in particular in mobile payments (Wang et al., 2021).

Many research studies today require more than two factors to ensure that impersonation cannot be successful. The three-factor method is deployed using traditional factors including *something you know, something you have*, and *something you are*, and it has been indicated that *something you have* is automatic for mobile users because the *something you have* is always with the user (the mobile device itself) (Bissada & Olmsted, 2017). Another study of blockchain-based e-voting on mobile devices recommends the use of the voter’s identification number (VIN), PIN, and OTP (Abayomi-Zannu, Odun-Ayo & Barka, 2019). In online banking, an OTP is generated from the registered IMEI (*something you have*) and is used as part of three factors of authentication (Shaji & Soman, 2017) in addition to a dynamically generated NFC code in a three-factor authentication, for example in an e-health context (Alghamdi, 2021). Symmetric keys can be used with other factors like passwords and biometric data to support a scheme for a system containing sensitive data (Liu et al., 2017). All three factors can be blended successfully in general, but cost effectiveness should be considered (Abiew, Jnr & Banning, 2020). In addition to the three classical factors, *somewhere you are* or location may be counted as the fourth factor (Choi & Zage, 2012). Low-cost locators such as Bluetooth and GPS can be implemented to track or locate a user physically while authenticating (Ramatsakane & Leung, 2017).

The more factors are used, the more confidence a system has; however, the usability aspect needs to be considered. People use these systems very often and cannot put effort into logging on or doing any complex authentication action several times a day (Sciarretta et al., 2018). A new multi-factor authentication scheme, SELAMAT, has been introduced to facilitate users’ access to cross-platform systems located in different geographical locations (Khalid et al., 2021). Similarly, Personal Identification Number (PIN) codes and OTP through a third-party authentication platform such as Google Authenticator can reduce users’ effort, promoting a single sign-on (SSO) experience (Sciarretta et al., 2018). In a security scheme focusing on usability and deployability, users do not need to memorize a password or token, instead using a smartphone to scan a dynamically generated Quick Response code (QR code) (Jindal & Misra, 2021). In terms of biometrics, it is perceived that smart biometrics can blend usability and security especially in mobile banking (Ndunagu & Nwoduh, 2019). Unlike traditional multi-factor authentication where it restricts users to using specific factors and they cannot choose which ones they prefer, *(t, n)* threshold authentication has been introduced to enable users to choose authentication factors based on their preference (Li et al., 2021). The use of Photo Response Non-Uniformity (PRNU) with face recognition shows better security level and better usability, as users do not need to memorize passwords or bring devices (Nimmy, Sankaran & Achuthan, 2018).

Numerous multi-factor authentication schemes have been proposed so far, but it has been reported that real and practical implementation is little, since they require too much effort from the user with a security level not as expected (Wang et al., 2020). Also, using some unfriendly, high-tech factors may inhibit use of those schemes because it requires a high user skill level, and authentication devices are not available everywhere (Ali et al., 2021). This is in line with a study (Das et al., 2019) revealing that more user-friendly multi-factor authentication is relatively essential, though very few existing papers focus on user evaluation.

A number of time attendance systems with security schemes for avoiding impersonation or spoofing have been proposed for different purposes. Most security schemes deploy biometrics, such as the real-time face detection-based approach (Kuang & Baul, 2020; Mady & Hilles, 2017; Srivastava et al., 2020; Shrestha et al., 2018; Kumar et al., 2020; Yusof et al., 2018) and fingerprints (Thejaswini et al., 2021; Hasan et al., 2020). Moreover, a number of studies have suggested hardware-based authentications as *something you have* such as RFID (Putrada & Abdurohman, 2020; Maramis & Rompas, 2018), Near-Field Communication (NFC) (Oo et al., 2018), and wireless sensor networks (Alassery, 2019), while location-based services for area restrictions have been used to track employees (Fatkharrofiqi et al., 2020) and students (Ding, Cao & Zhu, 2018). In terms of multi-factor attendance systems, a two-factor system using RFID and face identification has been implemented for employees (Kurniawan & Zaky, 2020). Another two-factor participant time attendance system deployed QR code identity and face verification as a contactless method during the COVID-19 situation (Pichetjamroen et al., 2021). A multi-factor method for student attendance uses face recognition with two different *somewhere you are* factors, including GPS and QR code (a student uses his/her mobile device to scan a QR code provided by a lecturer in a physical classroom) (Yazid et al., 2019). A multi-modal attendance tracking system uses three different *somewhere you are* factors, including GPS, WiFi location, and Wireless Local-Area Network (WLAN) location along with consideration of reliability aspects, such as the number of Bluetooth devices around the user and the sojourn time within a designated area (Liu et al., 2020). Despite the use of three location tracking methods plus two additional features, this scheme could be designated a single-factor method (*somewhere you are*) based on the classical factors. It can be seen that the majority of these schemes above use only a single factor, and there is little literature on multi-factor time attendance systems. The drawback of single factor is linked to the high possibility of impersonation, whereas the implementation of a multi-factor system is hardly usable despite higher security.

In real implementation today, many offices provide a time attendance system using single-factor or two-factor (*e.g*., magnetic card or/and biometric data like fingerprints or facial data). A single factor cannot confirm identity because people can intentionally or unintentionally use other credentials. A two-factor authentication is not practical due to time consumption, especially when involving biometric factors. Importantly, in the COVID-19 crisis, people feel inconvenienced when standing in long queues or contacting a time attendance machine. Increasing the number of machines does not make sense, especially for a large-scale organization like the government or an industrial park. A Bring-Your-Own-Device (BYOD) version or mobile version might have been proposed in order to transfer the costs to users and reduce health safety risks, but it deployed only one or two factors, which cannot confirm identity or avoid fraud, as mentioned earlier; increasing factors cannot confirm usability.

Moreover, some recent multi-factor authentication studies have presented new categorizations for each factor (Ali et al., 2021; Yazid et al., 2019; Liu et al., 2020) that are different from the traditional four factors emphasized in this paper; thus, these new, categorized factors can be overlapping in terms of the traditional categorization. Although the above studies have mentioned usability, the term is interpreted in different ways. In fact, usability could be considered as both tangible aspects and intangible aspects like speed, cost, user experience (or feeling), and law compliance while each proposed mechanism in existing studies does not cover all of these. Our previous work (Jaikla et al., 2020) confirmed the importance of the integration of all four factors in an attendance system. In this paper, we further that study by providing the technical details of process flows, design of all factors and how to blend them successfully, experiments and results, and emphasizing usability in all aspects above.

## Proposed comprehensive-factor authentication for a time attendance system and its experiments

This section aims to propose a usable time attendance scheme that deploys all three authentication factors, plus one location factor. Not only is security considered, but other usability aspects like ease of use, time taken, health safety, and privacy issues are also taken into account. Our proposed scheme is implemented in a mobile-based system or BYOD. The process is described in the scenario below.

### Registration process

Alice, a staff member at an R&D organization located in a technology park, has to register her account with our time attendance system for her first use. Her inputs with sensitive information (*e.g*., username and password) are required to confirm her identity. This sensitive information can be linked to the corporate Lightweight Directory Access Protocol (LDAP). In the meantime, her mobile device (hardware) is registered with our system as a user device. She then is required to take photos of herself or a selfie from different angles. These photos are stored in our system. The registration process is now complete, and her information can be updated at any time by the user. At the beginning of the registration process, the user is required to read our consent form based on the data privacy policy and choose whether to accept the privacy conditions.

### Authentication process

**Figure 1:**
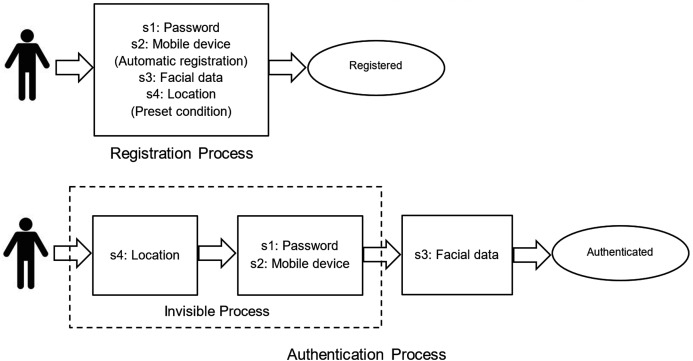
Registration and authentication scheme layout for user view.

Alice arrives at the R&D organization, which is situated in a technology park. Based on the organization’s human resources policy, she is allowed to check in anywhere in the technology park area or in any part of any building. She connects to the corporate WiFi, and then she is able to open our time attendance app. If she connects from any other WiFi network or mobile data (4G/5G), she will be rejected from the app (*somewhere you are factor*). If she is checking in for the first time, she has to input her username and password, which were previously registered, to verify her identify (*something you know factor*). The next time, she can choose to remember the password to bypass this process, as is seen in many other apps (*e.g*., Facebook). Also, if she opens the app from any other unregistered mobile devices, she will not be allowed to proceed (*something you have*). The next step is that she needs to scan her face, and the app detects and verifies only her face from her previously registered facial data (*something you are factor*). When this face verification is completed, the time attendance process is done. When looking at the first three factors above, all are verified simultaneously. If she checks in or out without any problem, she may feel as if the system verifies only *something you are* (face) because the other factors are automatically verified in the background, and the user may not be aware of this. The background process can be indicated as an “invisible” process. The user thinks that the total time consumed is only for face verification. This is what we expect, since it is not important whether a user is aware of the use of multiple factors. The scheme layout for user’s view is presented in Fig. 1. The symbol *s1*, *s2*, *s3* and *s4* in Fig. 1 represent *something you know*, *something you have*, *something you are*, and *somewhere you are*, respectively.

### System architecture

**Figure 2:**
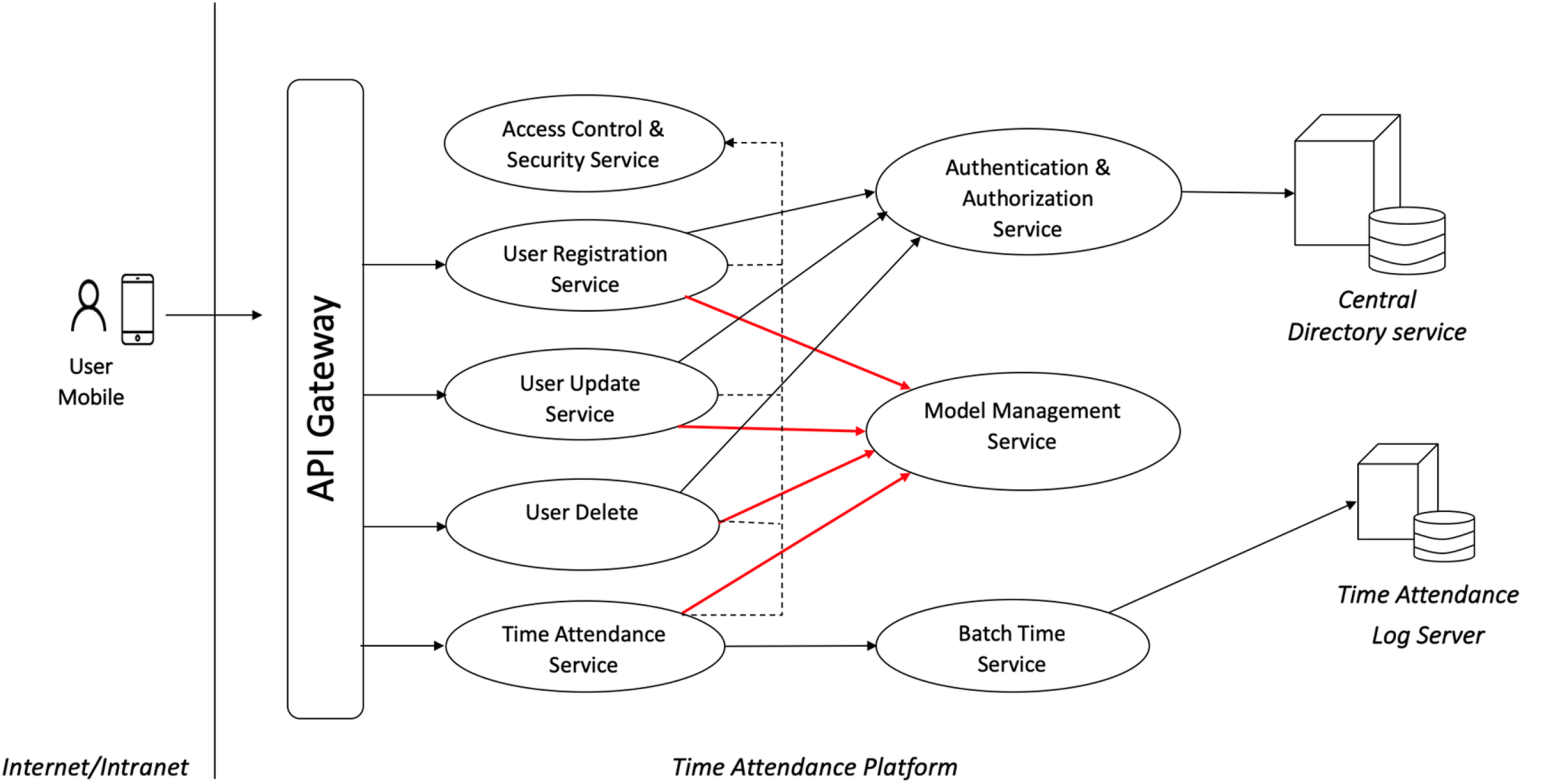
Overall system architecture of the comprehensive-factor time attendance service.

The time attendance system requires a mobile user to use our Application Programming Interface (API) to gain access to the services. The services include access control and security, registration, update, delete, time attendance, authentication and authorization, model management, and batch time services. The data storage units are a centralized directory unit and a time attendance data log unit. The overall system architecture of the service is displayed in Fig. 2 below.

The next sections are an example of the system design of all three plus one factors.

### Something you know

**Figure 3:**
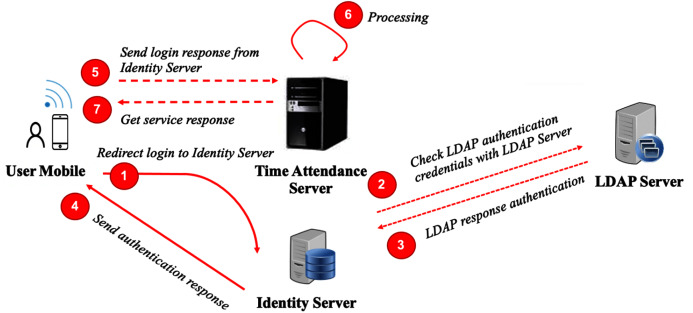
User authentication architecture.

Authenticating a user from something you know is first performed during the registration process, as mentioned earlier. A user is required to input a username and password, and these sensitive input data will be checked against the corporate account directory or LDAP. In this case, we used OpenLDAP (https://www.openldap.org/) as an account manager. This could be same as a single sign-on scheme for an organization’s intranet application or enterprise architecture, as depicted in Fig. 3. We used an identity server as a gateway, which is connected to the LDAP server to accept “calls” from several services, including our time attendance system.

**Figure 4:**
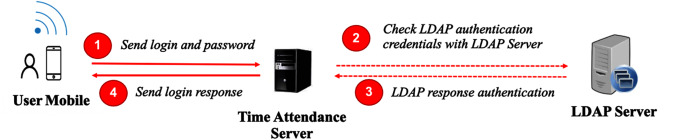
User authentication without an identity server.

Moreover, our scheme can work without an identity server. A user submits a username and password to the time attendance server, which is directly connected to an LDAP server. The LDAP server authenticates the credentials and responds as to whether the user is granted access to the system. This is displayed in Fig. 4.

The organization can benefit from using the existing account manager because there is no additional cost of managing accounts, and the security level of the account management or privilege management remains the same.

Moreover, if an organization accepts authentication through a third-party account platform such as Google or Facebook, this can also be used. After the first authentication, the user is allowed to have the app remember their password remembered; thus, the something you know factor can be bypassed the next time, similar to existing apps like Gmail or Facebook.

### Something you have

**Figure 5:**
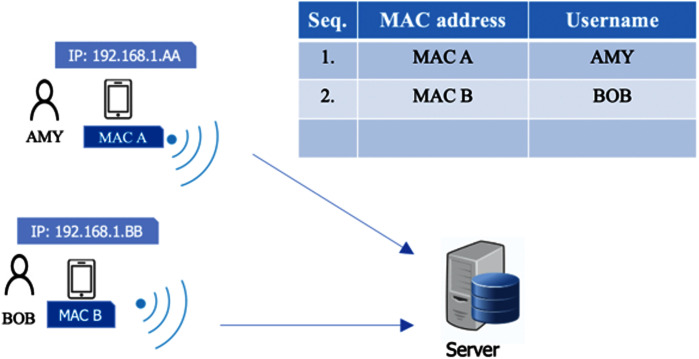
Device authentication (desired scenario).

**Figure 6:**
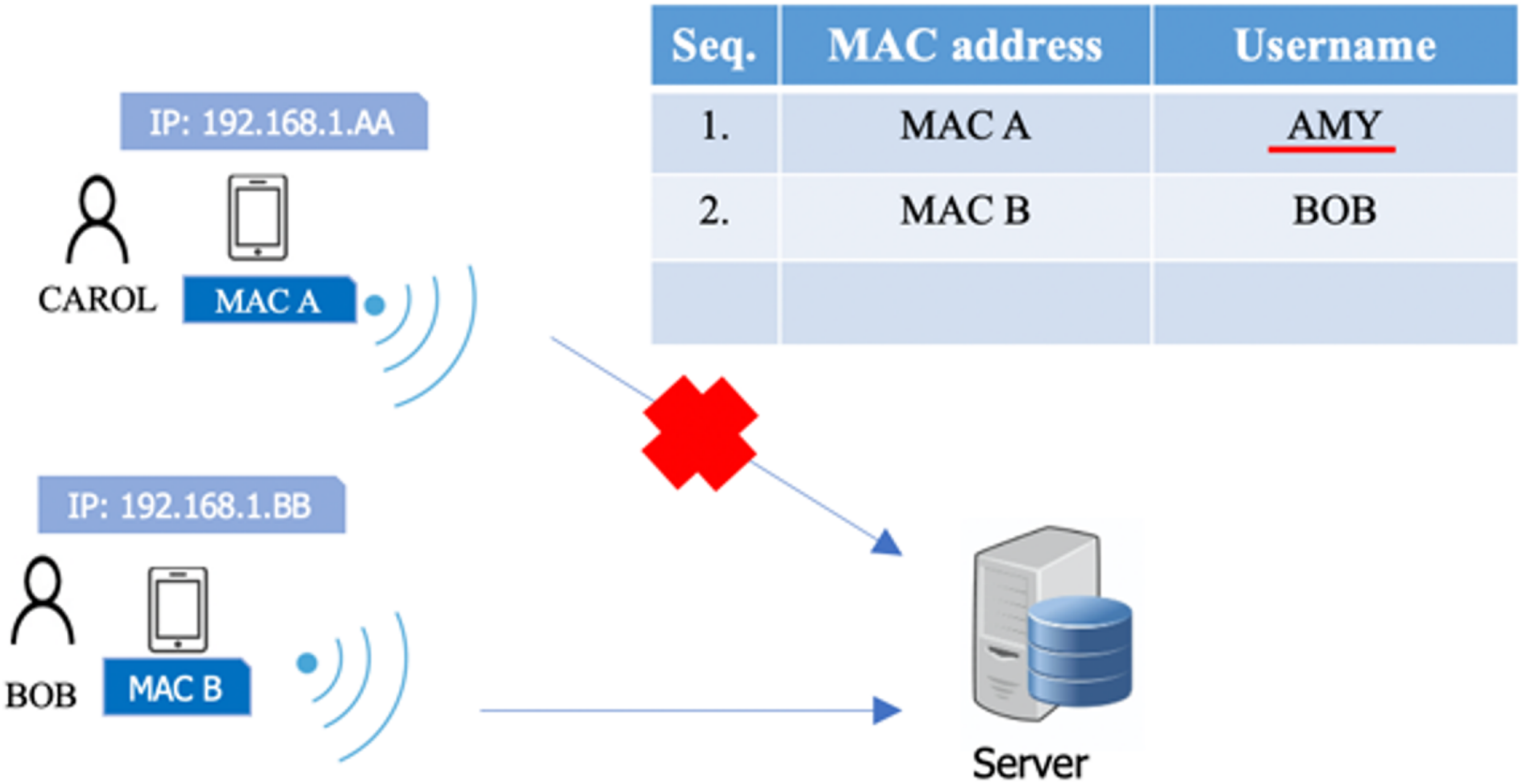
Scenario where a user checks in on own behalf.

**Figure 7:**
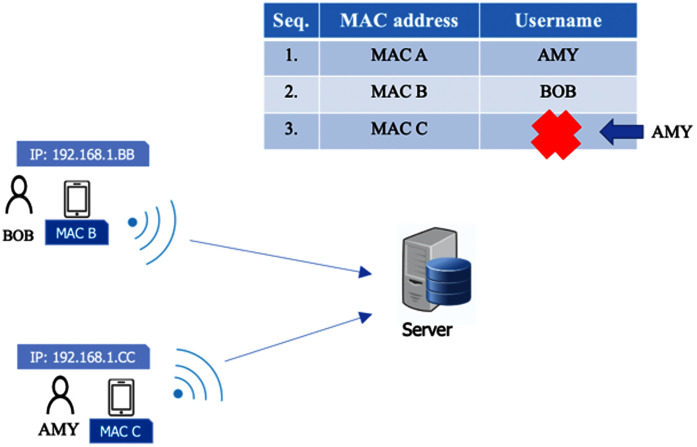
Scenario that a user uses other registered user’s device to check in for himself/herself.

Along with something you know, something you have is used to authenticate the owner of a mobile device. The system applies one user per device. This factor is deployed to avoid other users checking in or out on behalf of someone else in case the username and password are shared. In this case, we use an identification for the mobile device, such as the Media Access Control address (MAC address). This is because the wireless adaptor in each smartphone has a different MAC address, and each MAC address is unique. Therefore, MAC addresses can be used to identify a user (along with other factors). During a registration process in which a user authenticates with a username and password, information on the MAC address of the mobile device is stored in the time attendance server. Once a user accesses the system with a mobile device, the system can recognize a user from the MAC address immediately. In Fig. 5, Amy registers her device in the system. The MAC address (MAC A) of her device is also registered. Bob also registers his device which has a different MAC address (MAC B). In Fig. 6, if Amy gives her device to another user, Carol, asking her to check in on Amy’s behalf, Carol cannot do it because other factors such as the password and facial data (to be explained later) only belong to Amy. On the other hand, in Fig. 7, if Amy uses another registered user’s device (MAC C) to check in for herself, after she registers using her username and password, she will be rejected immediately because the MAC Address (MAC C) has been registered by the other user, and Amy must check in with her device (MAC A) due to the one-user-per-device requirement. Although MAC spoofing may be done successfully, a hacker needs to know a targeted MAC, and, more importantly, the hacker has to take too much effort to hack other factor as well, *e.g*., password, facial data and location.

### Something you are

Our scheme adopted a face verification technique to authenticate a user. Face verification is a one-to-one authentication in which the system knows the user in advance, and only then verifies whether the user is the right person. This biometric factor starts during the registration process. After a user registers with a username/password and mobile device (MAC address), the user is required to submit several selfie shots, as mentioned earlier. When the user wants to check in, he/she opens the app and then is authenticated by the other factors—*i.e*., username/password (remembered), device and location (described later), and finally his/her face. In this final step, the system recognizes the user in advance from the first two factors and therefore prepares the facial data of that user collected during registration to compare or verify with the live face. This step could take 1–2 s to respond with whether check-in is successful, with a timestamp. If not, the user needs to scan again or start the process again. In fact, a face recognition technique (many-to-many) can also be considered, but accuracy is lower than the face verification, and a false negative error is unacceptable in this scenario. Also, since this system deploys all factors, face verification is sufficient. In this section, we do not aim to present a new face verification or face recognition technique, because there are many existing techniques already available for free today. In this case, we used the Dlib library (http://dlib.net/). In our experience, we have tested up to 100 employees, and the accuracy level is 100% except in the case of identical twins (two twin employees). The detailed results will be presented in the next section. To avoid using the photo of a user’s face, face liveness detection by challenge questions such as blinking eyes, opening mouth, or smiling has been implemented. This can improve accuracy, but people are reluctant to do this every time they use the system, and the check-in process takes longer.

### Somewhere you are (location)

**Figure 8:**
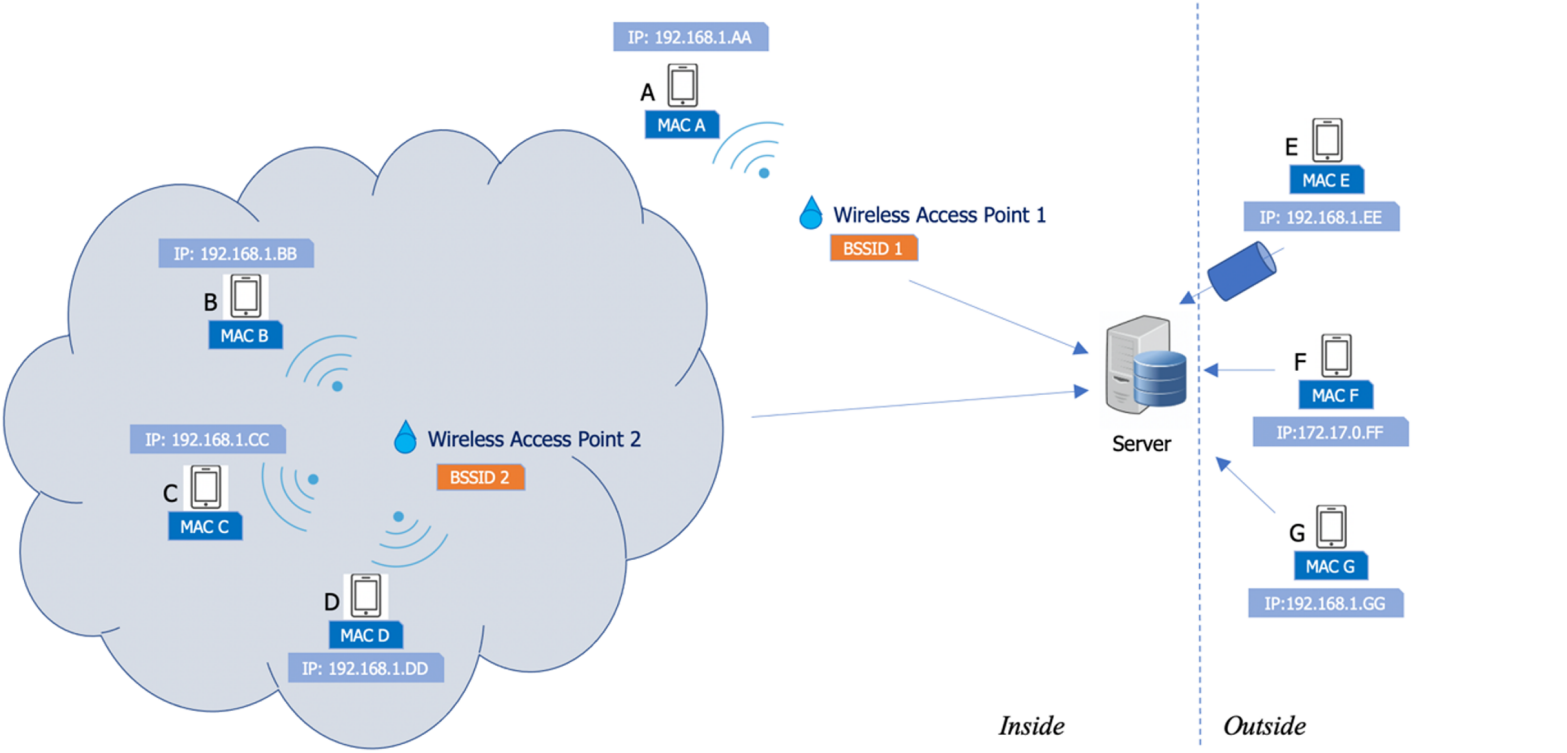
Verification of user location.

This location factor is detected in the very first stage. It is not a main factor, but it is used to support the other three factors. When a user opens the app, whether the user is located within the desired areas is checked immediately. This can be implemented in several ways, including GPS, Bluetooth, or Internet network. In this case, we chose the Internet network because it does not require additional hardware installation, nor do we need to ask users to enable the location service on their device. The system checks the Basic Service Set Identifier (BSSID) at a wireless access point. When a user’s device is connected to a corporate WiFi and opens the app, the system checks the BSSID at the device to determine if the device is connected to a desired wireless access point. Figure 8 demonstrates how the location factor is used in our scheme. In this case, some employees (device A) are allowed to check in at any part of the office area, whereas others (device B, C, and D) are required to check in at only specific areas such as a specific floor or room in a building. As a result, the employees in the first group can use the app with their device and connect to any wireless access point at an office, while the other group can use the app only when connected to a specific wireless access point.

A device connected from an outside network is also not allowed (device E, F, and G). In addition to BSSID, the system also checks the IP address of a user’s device, which must be within the range of IP addresses in the office (such as 192.168.1.xx). Also, the range of IP addresses must not include IP addresses for VPNs, which do not allow checking in from the outside. Thus, devices E and F are not allowed because their IP addresses are not within range. A device connected from an outside network (device G) may obtain an IP address that appears similar to an IP address in the office (192.168.1.GG) or, in another case, an IP address is spoofed. Hence, the system needs to check remote addresses in addition to IP addresses in the office. In this case, the remote address of device F is different in the range, and therefore connection from device F is not permitted.

## Results

The results were partly presented in the previous section. In this section, we will present the overall results. One hundred employees at the technology park were involved in the experiment. These employees were selected on a voluntary basis and were required give consent because the system collects personal data such as facial data and location. Therefore, the samplings cannot be categorized by age and gender. However, all participants had good digital literacy, using mobile devices and the Internet on a regular basis. All participants were between 20 to 60 years old, which is the typical age of employees at this organization. We tested all four factors for the security aspect and usability aspect based on the scenario mentioned in the previous section. The details of the results are presented in factor categories. Employee’s identities (ID 001-100) presented in this experiment are pseudonymized. The results focus on accuracy in different perspectives.

### Something you know

The results show accuracy when employees log in through our system. Accuracy means whether the results of their attempts to login in to our system are similar to the results of their attempts to login to the organization’s existing Intranet (LDAP). Participants were asked to make an intention for a successful login or login failure (due to invalid password) to confirm that in this factor, a negative error and positive error will not occur. The results in Table 1 show that in this *something you know* factor, all sampled employees logged in correctly, which is in line with the accuracy of existing Intranet login on regular use.

**Table 1:** The results of the *something you know* factor.

Employee ID	Intend to succeed in logging	Intend to fail in logging	Overall accuracy
001–100	Accurate (successful)	Accurate (access denied)	Accurate

### Something you have

The results show an accuracy of situation (a) when an employee uses their own registered device to check in, and (b) when an employee uses another device (unregistered device or device registered by different person) when checking in. This is to check whether spoofing can be done successfully when a person uses another employee’s mobile phone to check in on their behalf. The results in Table 2 show that in this *something you have* factor, in the desired situation, all sampled employees passed this factor accurately when using their own device, whereas all were rejected when using another device that was not their own registered device.

**Table 2:** The results of the *something you have* factor to verify whether an employee uses own device.

Employee ID	Use registered own device	Use another device	Overall accuracy
001–100	Accurate (successful)	Accurate (access denied)	Accurate

While it is assumable that the MAC can be spoofed, a hacker can get initial access only in case they knew the target MAC (registered device). Also, they need to know the target’s password. If the hacker fails to obtain one of the data, their access will be denied immediately. The results below show an attempt to access the system using a MAC-spoofing device, where (a) the spoofing MAC is registered and (b) the spoofing MAC is not registered, and the hacker does not know the target’s password. In this experiment, we divided employees into two groups: the first group was the first 50 employees whose employee ID was spoofed and a hacker did not know the MAC’s target; the second group is the remaining 50 employees whose employee ID was spoofed and their MAC was known and spoofed. The results in Table 3 show that in the first group, if a hacker can spoof any MAC address but the hacker still does not know the target employee’s MAC address (or employee device), the access will be denied in all 50 samplings. Also, if a hacker knows the target employee’s MAC address and can spoof it, the hacker can pass through this factor, but eventually the hacker cannot pass through the something you know factor in all other 50 samplings because the hacker does not know the employee’s password. In addition, the hacker cannot pass through the *something you are* (facial data) factor and perhaps *somewhere you are* (location) factor in cases where access to corporate WiFi requires a password from LDAP.

**Table 3:** The results of the *something you have* factor.

Employee ID who is spoofed	Do not know the MAC’s target	Know the MAC’s target	Overall accuracy
Not identified #1–#50	Accurate (access denied)	N/A	Accurate (access denied)
001–050	N/A	Accurate (initial access allowed)	Accurate (access denied due to invalid password)

### Something you are

The results in Table 4 show the accuracy of the results when an employee scans their face after passing the two previous factors. It is noted that Employee ID 089 and 098 are the identical twins that our face verification mechanism (mentioned in the previous section) was unable to differentiate. Therefore, the overall accuracy is 98%, while the negative error rate is 2%. However, in a real situation, it is unusual that these twins would share a password or share a device for the whole day; thus, they will not gain an access or cannot check-in on behalf of each other successfully.

**Table 4:** The results of the *something you are* factor.

Employee ID	Use own face	Use another face	Overall accuracy
001–100 (except 089 and 098)	Accurate (successful)	Accurate (access denied)	Accurate
089 (identical twin to 098)	Accurate (successful)	Inaccurate (access allowed)—using the face of ID 098	In accurate
098 (identical twin to 089)	Accurate (successful)	Inaccurate (access allowed)—using the face of ID 089	In accurate

### Somewhere you are (location)

The results in Table 5 show the accuracy of the scenario when an employee accesses the system within a designated area, outside the corporate WiFi, and physically outside the organization *via* a corporate Virtual Private Network (VPN). All samplings can get access when they are within a designated area or corporate WiFi range, but their access is denied if they are outside the corporate WiFi range or they connect to the system *via* a corporate VPN. In a real situation, this location factor is the first step of the authentication process because the user’s location is immediately verified shortly after opening the app.

**Table 5:** The results of the *something you are* factor.

Employee ID	Within a designated area	Outside corporate WiFi	*Via* a corporate VPN	Overall accuracy
001–100	Accurate (successful)	Accurate (access denied)	Accurate (access denied)	Accurate

### Overall results

The results above have confirmed the accuracy of our proposed scheme in a simulated real environment. Only the case of identical twins in the *something you are* factor showed inaccurate results, but in fact it is impossible that the twins could or would share all the remaining factors with each other in the real use of time attendance. In addition, the authentication process was done simultaneously and automatically for each factor except the *something you are* factor. Thus, the time taken to check-in for each employee was very short and depends only on the *something you are* factor. The first three-factor (*somewhere you are*, *something you know*, and *something you have*) authentication process is automatic and normally take less than one second in all samplings. The last process using facial data (*something you are*) takes approximately one second to detect and verify the face. Therefore, the overall time taken is less than two seconds. This user-friendly scenario encourages employees to use it, since they only feel authenticated by face verification as the other three factors are automatically authenticated as opposed to existing systems that require several actions from the user. In terms of attempt to spoof, the results above show the possibility for spoofing one factor, but in a real situation, it takes too much effort to spoof all four factors. The summarized results are presented in Table 6.

**Table 6:** The overall results of the experiment.

Factors/attributes	Results
*Something you know* (Username/Password)	100% Accurate (similar to our existing Intranet system)
*Something you have* (Device)	100% Accurate (once a device is registered, other user cannot use the registered device on behalf)
*Something you are* (Face verification)	98% Accurate (two persons who are twin cannot be differentiated)
*Somewhere you are* (Location)	100% Accurate (employees who are outside the desired area including a location outside the technology park, not on the desired floor at the desired building and through VPN)
Average time used to check-in	Less than two seconds
Attempt for spoofing	Attempt for spoofing is possible, and it depends on the security strength of each factor. In our experiment, MAC spoofing be done in general as mentioned earlier. However, the hacker does not know the target’s MAC unless it is willingly given by the targeted user. However, the hacker still needs to acquire the target’s identity of the other factors, and has to borrow the target’s device. In this scenario using BYOD, people today feel reluctant to lend their own mobile device, even for a short time.

## Discussion

The comparison of our proposed scheme with other existing systems is discussed in Table 7. It is confirmed that our proposed scheme, when used in a comprehensive-factor time attendance system, has the largest number of factors (four), high security level (due to four factors), high accuracy level (based on the results), least action required (one if an action for tapping the app icon is counted, zero if not counted), and the shortest time taken for authentication (less than two seconds). This is opposed to most existing schemes where more factors are used, more actions and efforts required, and usability decreasing. Thus, security level and accuracy level in those schemes cannot confirm usability in those existing studies. It is noted that the schemes and systems used in the comparison include multi-factor authentication studies, frameworks, and concepts in general, and specific attendance systems. Number of factors means the four factors used in this paper, including s1 for *something you know*, s2 for *something you have*, s3 for *something you are*, and s4 for *somewhere you are*. Security includes medium (two factors), high (more than two factors and one of them is biometrics or onsite verification by human), and N/A (one factor). Accuracy includes medium (no biometrics factor included) and high (biometrics factor included or multiple same factors). Actions required means the number of actions (*e.g*., clicking, tabbing, and typing) a user needs to take from start to end in an authentication process. Speed is time taken from start to end.

**Table 7:** The comparison between our proposed scheme and existing studies.

Schemes/systems	Number of factors	Security	Accuracy	Actions required	Speed
Mobile voting framework (Abayomi-Zannu, Odun-Ayo & Barka, 2019)	2 (s1,s2)	Medium	Medium	At least 2	N/A
Multi-factor authentication protocol based on fuzzy extractor (Mohammed & Yassin, 2019)	2 (s1,s3)	Medium	High	2	N/A
Multi-factor authentication with single sign-on (Sciarretta et al., 2018)	2 (s1,s2)	Medium	Medium	2	N/A
Multi-factor authentication using mobile app and camera (Jindal & Misra, 2021)	2 (s2,s3)	Medium	Medium to high	2	N/A
Multi-factor authentication with location security (Ramatsakane & Leung, 2017)	3 (s1,s2,s4)	Medium	Medium to high	At least 2	Minimum of 5 s
Multi-factor authentication for net banking (Shaji & Soman, 2017)	2 (s1,s2 or s2,s3)	Medium	Medium to high	2	N/A
Three-factor authentication concept (Bissada & Olmsted, 2017)	3 (s1,s2,s3)	High	High	2 or 3 (not stated)	N/A
Three-factor authentication in ATM (Abiew, Jnr & Banning, 2020)	3 (s1,s2,s3)	High	High	3	N/A
Three-factor authentication in e-health (Alghamdi, 2021)	3 (s1,s2,s3)	High	High	3	N/A
Employee attendance system (Kurniawan & Zaky, 2020)	2 (s2,s3)	Medium	High	2	N/A
Participant time attendance system (Pichetjamroen et al., 2021)	2 (s2,s3)	Medium	High	2	N/A
Four-factor student attendance system (Yazid et al., 2019)	2 (s3,s4 based on the classical factors in this paper)	High (due to onsite verification by human)	High	4	N/A
Multi-modal attendance tracking system (Liu et al., 2020)	1 (s4, based on the classical factors in this paper)	N/A	High (for location)	N/A	N/A
Comprehensive-factor time attendance system (the proposed scheme)	4 (s1,s2,s3,s4)	High	High	1 (or zero)	Less than 2 s

In terms of security, our scheme applies all authentication factors to ensure that spoofing of all factors does not occur. In other words, in this case study, no one can check in/check out on behalf of another, compared to the existing time attendance schemes. The accuracy of the authentication of each factor depends on the mechanism or algorithm developed to verify each factor. For example, the accuracy of face verification depends on the AI model used. More than 100 people, including those who checked in from outside networks and VPNs, were tested in a real environment in the technology park in all factors, and there was no error rate in all factors except false negatives in *somewhere you are* (facial data) in the case of identical twins, as mentioned above. It is not surprising that only *somewhere you are* showed a false negative error, but the other factors worked correctly, and checking in on another’s behalf thus does not occur. According to our users’ feedback, some users raised the concern that someone (Alice) who is absent today may be able to give her mobile phone to her colleague (John) to check in on her behalf, and John can use a photo of Alice to verify facial data (in case the face verification algorithm cannot differentiate between an actual face and a photo of a face). In fact, live face detection used to be applied to reduce this problem, but we found that this takes a much longer time, and users find it difficult and embarrassing to interact with the liveness function every day; thus, it was suggested that we remove this function. This scenario is unlikely to arise today because people do not share their devices with others, even with family members. This also includes the case of identical twins who do not share a device with each other. Therefore, to break the system successfully, all factors must be hacked at the same time in a real implementation.

As mentioned earlier, the study did not attempt to propose a novelty of security mechanisms of “each” factor, but rather presents a scheme of how to integrate all of them successfully in a real environment. Some of toolkits such as Dlib for facial data exist, but we demonstrated that using face verification (one-to-one) is enough because the system has already recognized a username (who is using), which is better than face recognition (many-to-many) in terms of accuracy and usability. A practitioner can use other face verification mechanisms. In addition, if one of each factor is spoofed (such as MAC), a hacker still needs to hack three other factors in order to spoof successfully.

In addition to security issues, the evaluation in this study is also based on important criteria of authentication on a mobile device, including time taken, impact of the user actions required, and user reactions to the methods (Trewin et al., 2012), as well as usability issues like ease of use, health safety, and legal matters (Vorakulpipat, Pichetjamroen & Polprasert, 2021). The system verifies all three plus one factors while the user feels that they were required to take only one action (face verification). The time taken for the entire authentication process in our system is similar to the time taken just for face verification because the other factors are verified in the background. This normally takes less than two seconds. An organization that previously used a single-factor or two-factor system would not see any major change in terms of time taken or effort a user needs to make. Therefore, time taken here means only a “short time” but also includes a number of actions a user needs to take. One aspect that emerged from the study is a seamless process that confirms usability in a security implementation. The term usability also includes this “seamless” factor by which a user feels “smooth” as if they are not being validated by several authentication mechanisms. In this case, everyone except IT specialists realized that the system uses only one factor (facial data), since only this factor is visible and needs interaction (Vorakulpipat, Pichetjamroen & Polprasert, 2021), while the authentication of the rest of the factors looks “invisible.” Of equal importance, a seamless process in this case is the result of a trade-off between usability and security (Allen & Komandur, 2019).

In terms of health safety, a user uses his/her own device or BYOD, not sharing the device with others. This could be regarded as a contactless scheme, since a user does not contact another’s device. Additionally, they do not need to wait in a long queue compared to physical time attendance systems. It was confirmed in Marks et al. (2021) that digital transformation during and post-COVID-19 should be carefully done, since there are a number of information technology (IT), security, and health safety challenges, but people are not aware of this holistic vision. In the final step, a user will give their consent related to data privacy. The user has the right to update or delete their facial data at any time, and the system applies “the right to be forgotten” according to data privacy law. Data subjects have expressed the concern that using too many factors (especially facial data) to check into an organization seems unnecessary, and the use of data should be reasonable, as confirmed in Singh & Cobbe (2019). Therefore, this system is only optional. In this case study, our proposed scheme does not aim to replace the existing system. The physical system that uses single-factor (*e.g*., magnetic card) identification still exists for anyone who does not have a smartphone or who does not accept the terms and conditions of data protection and data privacy.

## Conclusions

This paper presented an authentication scheme using all possible factors including three classical factors and an additional location factor. This scheme was applied to a mobile-based time attendance system, and the system shows that using all four factors can confirm a high level of security and a low possibility of spoofing attempts. Besides the security advantage, the system developed usability with seamless user interactions while being authenticated. A user is required to take only one action to pass all four factors in a short time. The proposed scheme in this paper demonstrated a possible way to increase authentication factors while maintaining usability, and user involvement did not increase, as opposed to other existing security systems. Focusing on the security accuracy of each factor may not be practical unless usability is not a concern. Finally, the use of one’s own device or BYOD, contactless interaction (not contacting other person’s device), and least user action required is aimed at promoting health safety during the pandemic situation. Legal issues related to data privacy have been also raised as important concerns when adopting an access control system.

To further the study, our proposed schemes can be extended to a service platform *via* API or a library where other organizations are able to develop their own comprehensive-factor time attendance system. The number of factors to be verified is flexible and can be adjusted depending on the security level needed. This flexibility is similar to the security level of each factor, as seen previously in the location verification in the technology park. Another example of this adjustment is that if this scheme is applied to employees working from home, IP addresses for the corporate VPN can be allowed for check-in.

## Supplemental Information

10.7717/peerj-cs.678/supp-1 Supplemental Information 1 Source code of our application for mobile devices. The name of the application is AtTime.

10.7717/peerj-cs.678/supp-2 Supplemental Information 2 The results of accuracy of our proposed schemes in terms of accuracy of each factor in different situations.

## Additional Information and Declarations

### Data Availability

The following information was supplied regarding data availability: The raw measurements and our source code are available in the Supplementary File.
