# Supplementary figures and images for: Usable comprehensive-factor authentication for a secure time attendance system
Source: PeerJ Comput Sci. 2021 Aug 16;7:e678. doi: 10.7717/peerj-cs.678 (PMC8384039; doi:10.7717/peerj-cs.678)

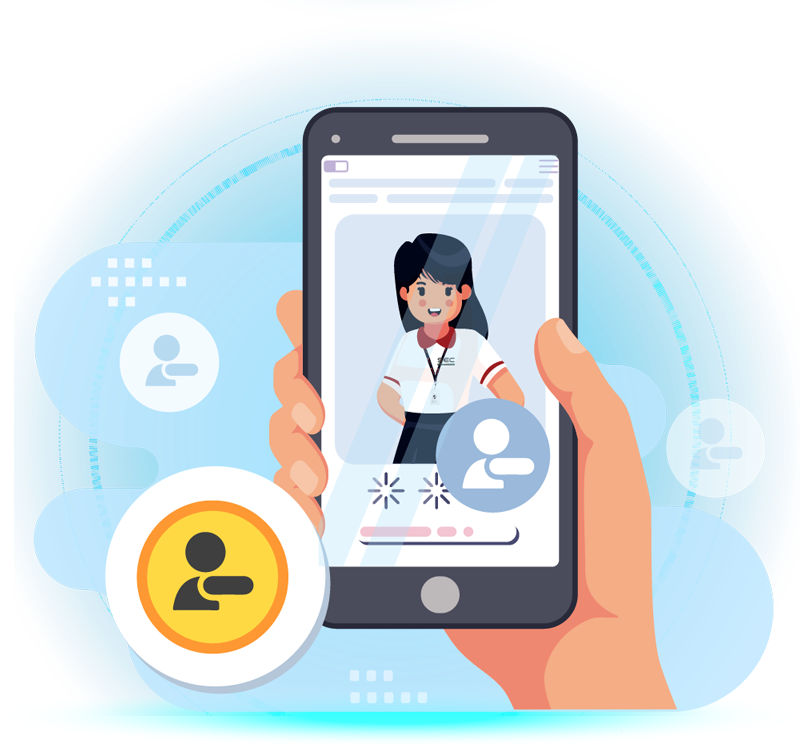

Supplement: Supplemental Information 1 [file peerj-cs-07-678-s001.zip › TIME/TIME/Assets.xcassets/i_nectec_remove_logo.imageset/i_nectec_remove_logo.png]

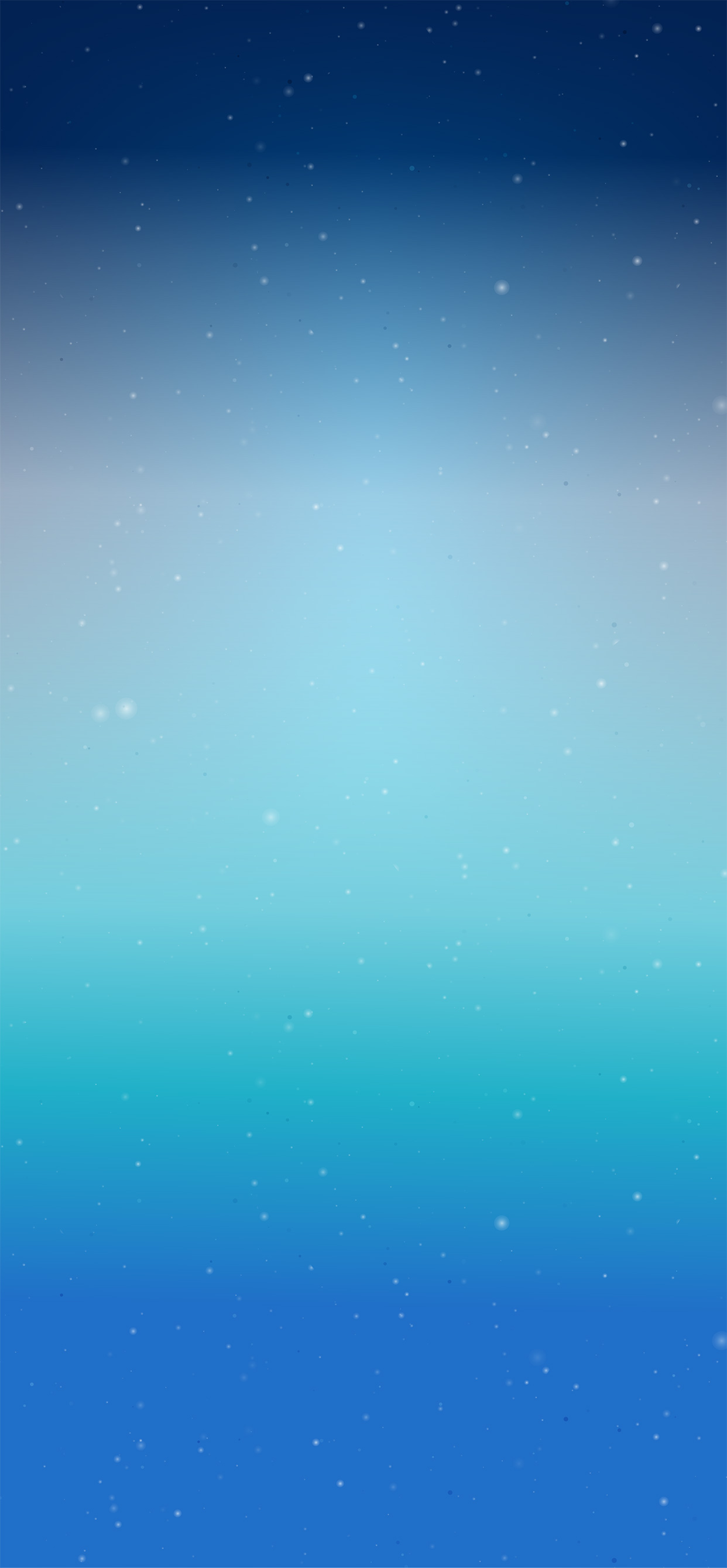

Supplement: Supplemental Information 1 [file peerj-cs-07-678-s001.zip › TIME/TIME/Assets.xcassets/bg.imageset/bg.jpg]

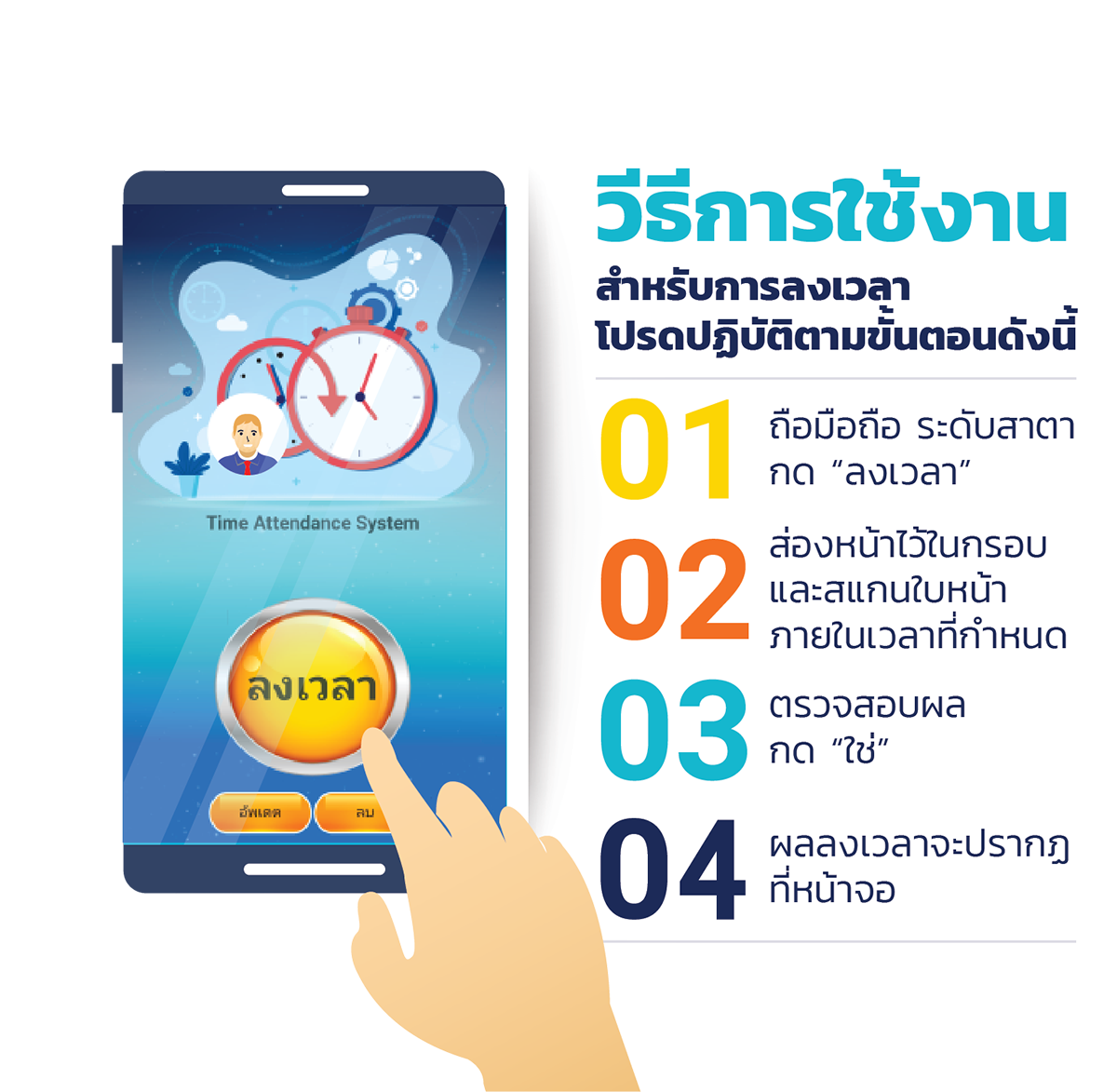

Supplement: Supplemental Information 1 [file peerj-cs-07-678-s001.zip › TIME/TIME/Assets.xcassets/i_nbtc_checkin_howto.imageset/i_nbtc_checkin_howto.png]

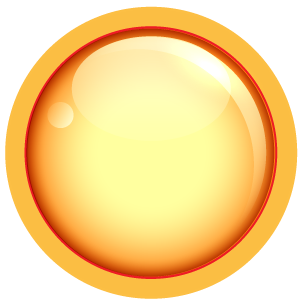

Supplement: Supplemental Information 1 [file peerj-cs-07-678-s001.zip › TIME/TIME/Assets.xcassets/b_checkin_btn_pressed.imageset/b_checkin_btn_pressed.png]

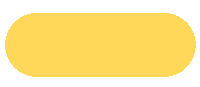

Supplement: Supplemental Information 1 [file peerj-cs-07-678-s001.zip › TIME/TIME/Assets.xcassets/b_main_btn_pressed.imageset/b_main_btn_pressed.png]

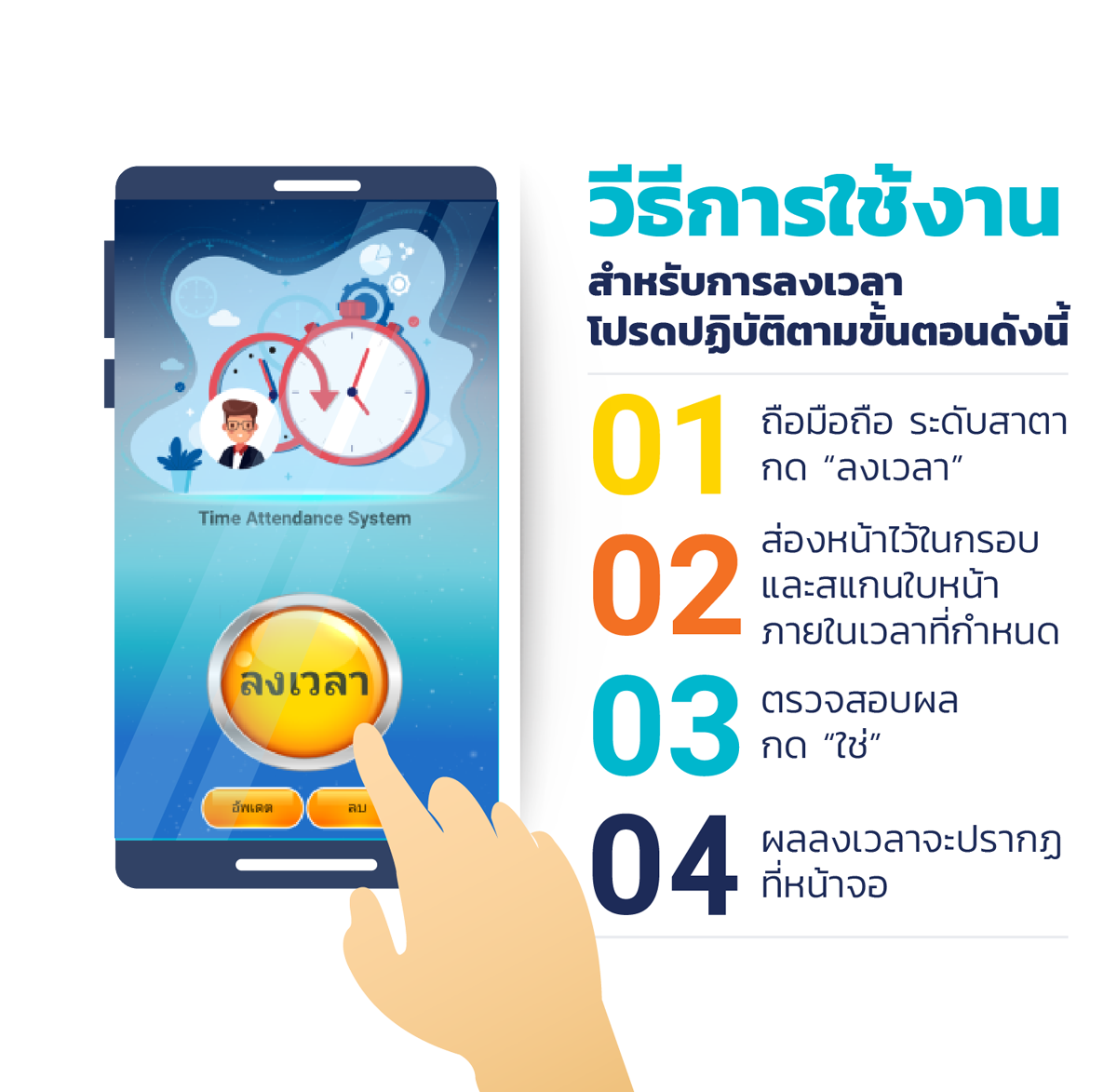

Supplement: Supplemental Information 1 [file peerj-cs-07-678-s001.zip › TIME/TIME/Assets.xcassets/i_nectec_checkin_howto.imageset/i_checkin_howto.png]

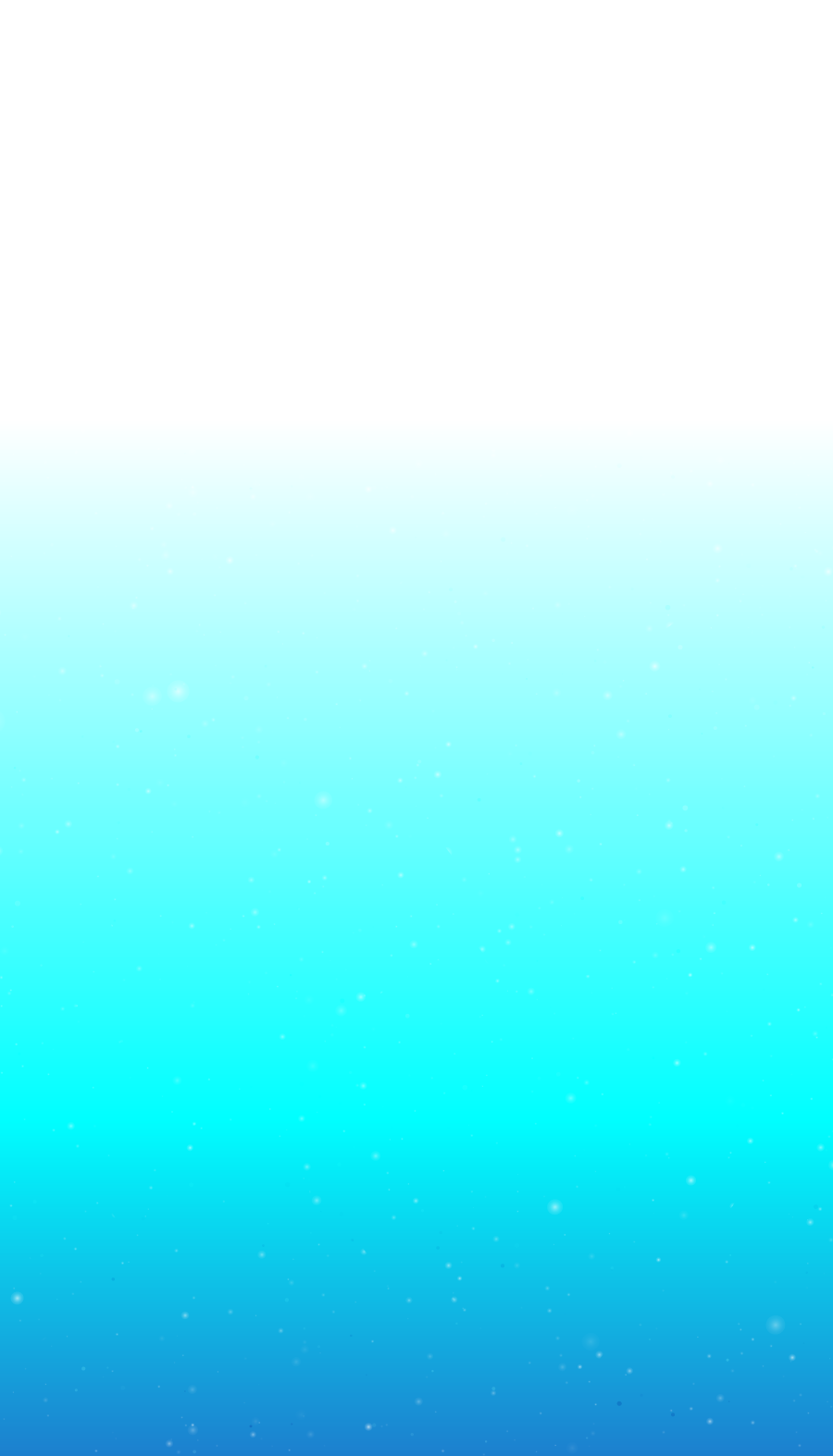

Supplement: Supplemental Information 1 [file peerj-cs-07-678-s001.zip › TIME/TIME/Assets.xcassets/bgAgreement.imageset/bg.png]

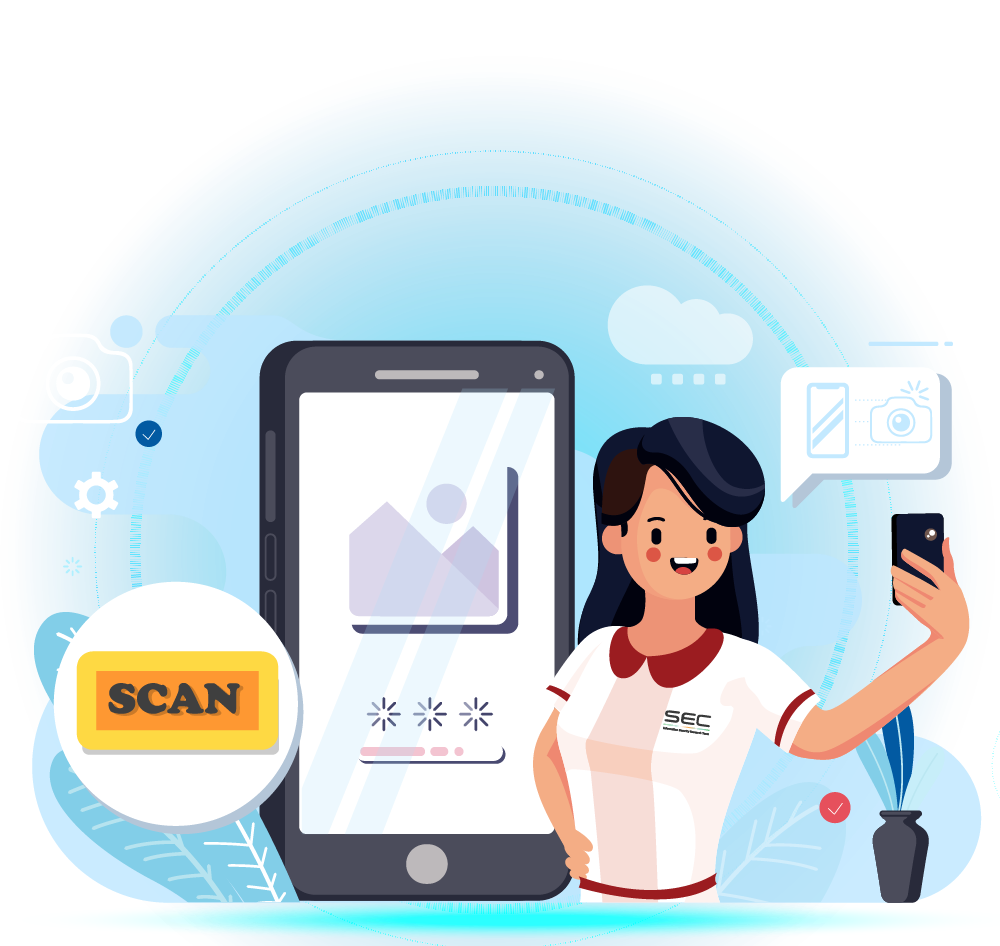

Supplement: Supplemental Information 1 [file peerj-cs-07-678-s001.zip › TIME/TIME/Assets.xcassets/i_nectec_capture_prepare.imageset/i_main_logo3.png]

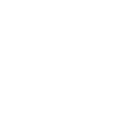

Supplement: Supplemental Information 1 [file peerj-cs-07-678-s001.zip › TIME/TIME/Assets.xcassets/b_close.imageset/b_close.png]

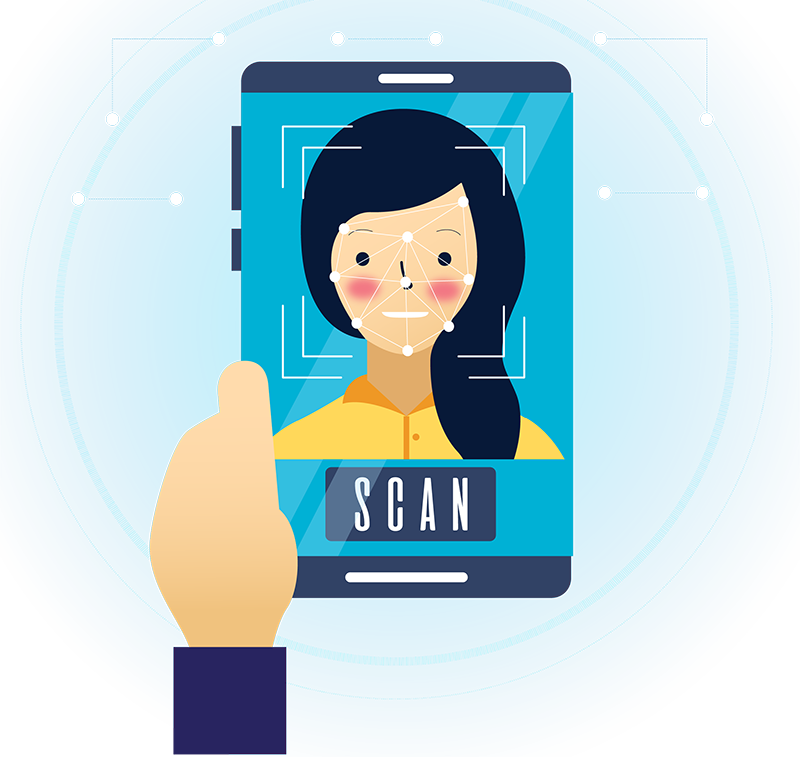

Supplement: Supplemental Information 1 [file peerj-cs-07-678-s001.zip › TIME/TIME/Assets.xcassets/i_nbtc_capture_prepare.imageset/i_nbtc_capture_prepare.png]

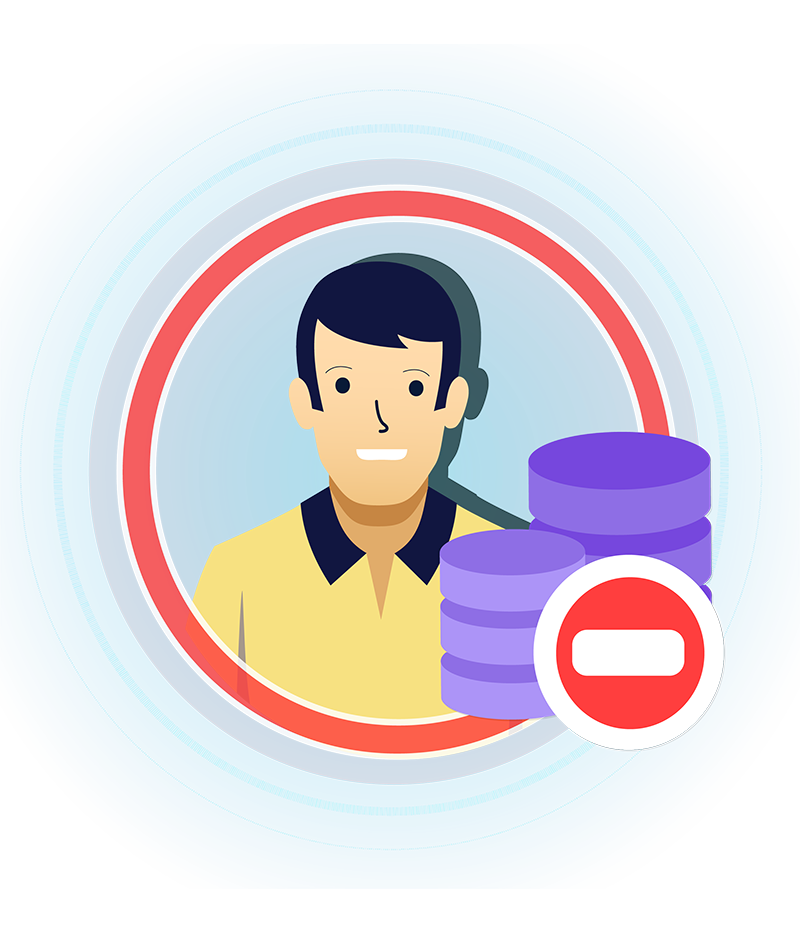

Supplement: Supplemental Information 1 [file peerj-cs-07-678-s001.zip › TIME/TIME/Assets.xcassets/i_nbtc_remove_logo.imageset/i_nbtc_remove_logo.png]

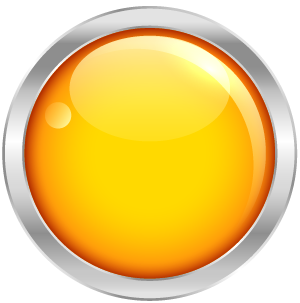

Supplement: Supplemental Information 1 [file peerj-cs-07-678-s001.zip › TIME/TIME/Assets.xcassets/b_checkin_btn_normal.imageset/b_checkin_btn_normal.png]

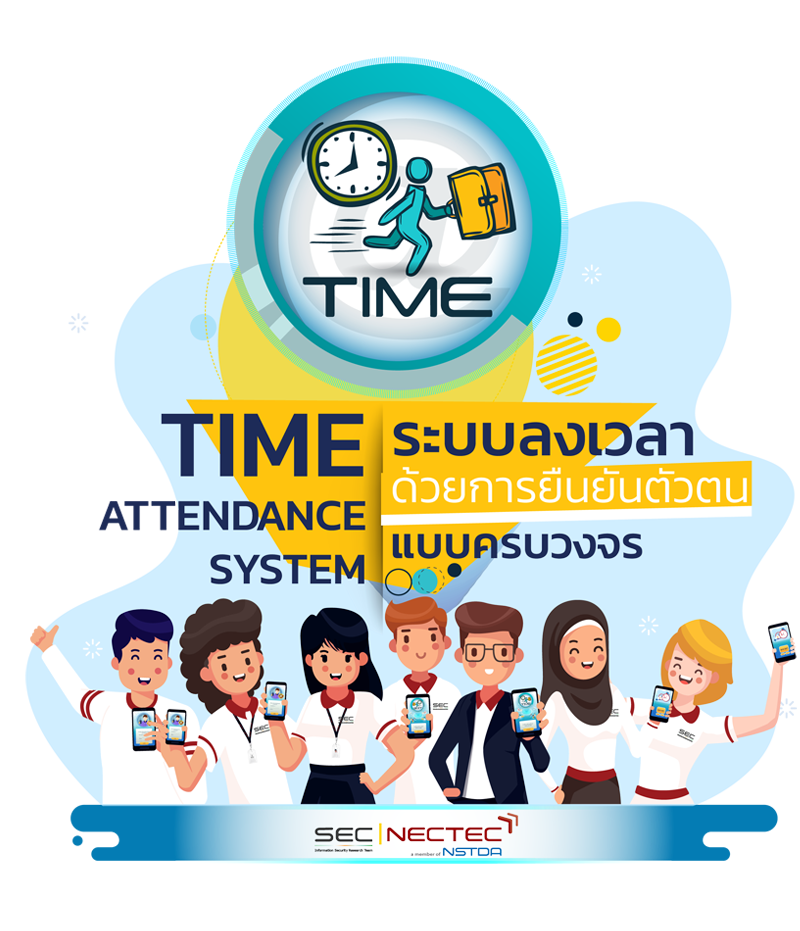

Supplement: Supplemental Information 1 [file peerj-cs-07-678-s001.zip › TIME/TIME/Assets.xcassets/i_nectec_main_logo1.imageset/i_nectec_main_logo1.png]

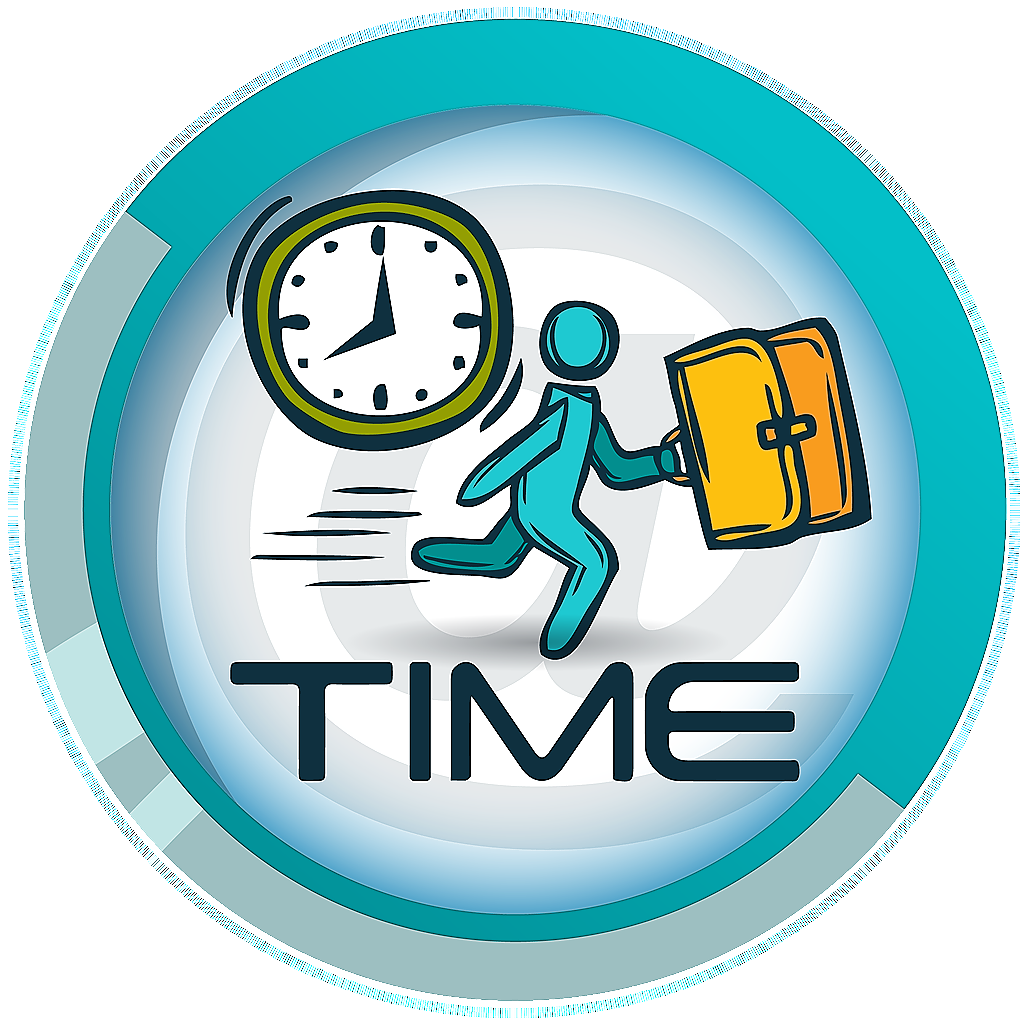

Supplement: Supplemental Information 1 [file peerj-cs-07-678-s001.zip › TIME/TIME/Assets.xcassets/AppIcon.appiconset/1024.png]

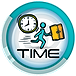

Supplement: Supplemental Information 1 [file peerj-cs-07-678-s001.zip › TIME/TIME/Assets.xcassets/AppIcon.appiconset/76.png]

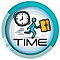

Supplement: Supplemental Information 1 [file peerj-cs-07-678-s001.zip › TIME/TIME/Assets.xcassets/AppIcon.appiconset/60.png]

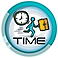

Supplement: Supplemental Information 1 [file peerj-cs-07-678-s001.zip › TIME/TIME/Assets.xcassets/AppIcon.appiconset/58.png]

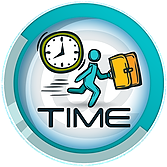

Supplement: Supplemental Information 1 [file peerj-cs-07-678-s001.zip › TIME/TIME/Assets.xcassets/AppIcon.appiconset/167.png]

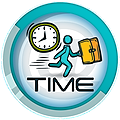

Supplement: Supplemental Information 1 [file peerj-cs-07-678-s001.zip › TIME/TIME/Assets.xcassets/AppIcon.appiconset/120-1.png]

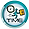

Supplement: Supplemental Information 1 [file peerj-cs-07-678-s001.zip › TIME/TIME/Assets.xcassets/AppIcon.appiconset/29.png]

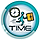

Supplement: Supplemental Information 1 [file peerj-cs-07-678-s001.zip › TIME/TIME/Assets.xcassets/AppIcon.appiconset/40-1.png]

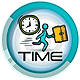

Supplement: Supplemental Information 1 [file peerj-cs-07-678-s001.zip › TIME/TIME/Assets.xcassets/AppIcon.appiconset/80-1.png]

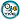

Supplement: Supplemental Information 1 [file peerj-cs-07-678-s001.zip › TIME/TIME/Assets.xcassets/AppIcon.appiconset/20.png]

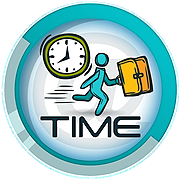

Supplement: Supplemental Information 1 [file peerj-cs-07-678-s001.zip › TIME/TIME/Assets.xcassets/AppIcon.appiconset/180.png]

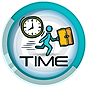

Supplement: Supplemental Information 1 [file peerj-cs-07-678-s001.zip › TIME/TIME/Assets.xcassets/AppIcon.appiconset/87.png]

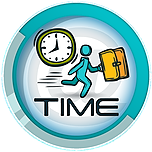

Supplement: Supplemental Information 1 [file peerj-cs-07-678-s001.zip › TIME/TIME/Assets.xcassets/AppIcon.appiconset/152.png]

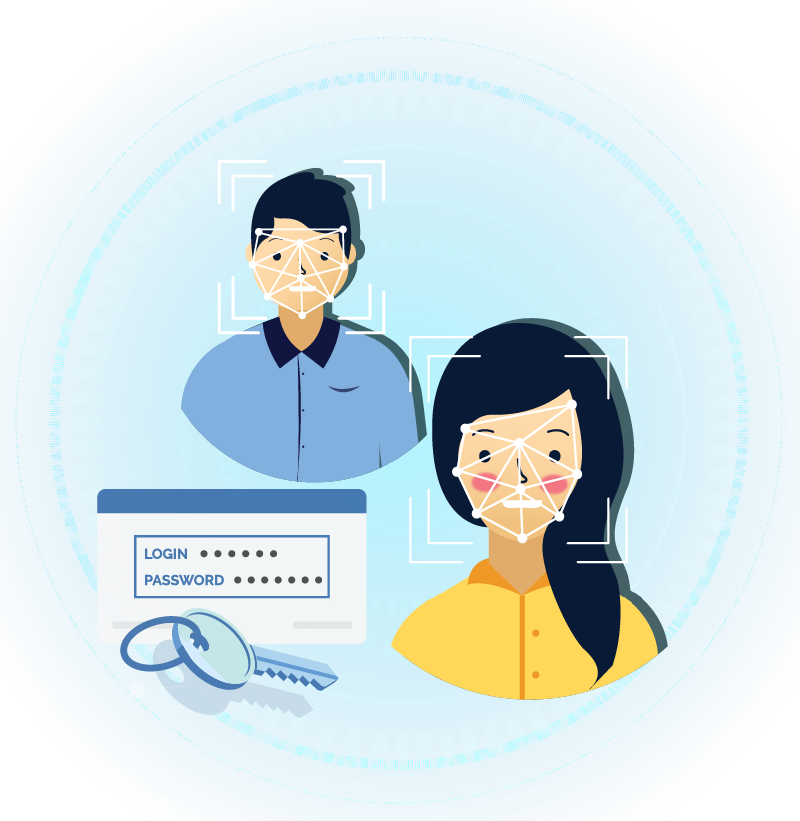

Supplement: Supplemental Information 1 [file peerj-cs-07-678-s001.zip › TIME/TIME/Assets.xcassets/i_nbtc_main_logo2.imageset/i_nbtc_main_logo2.png]

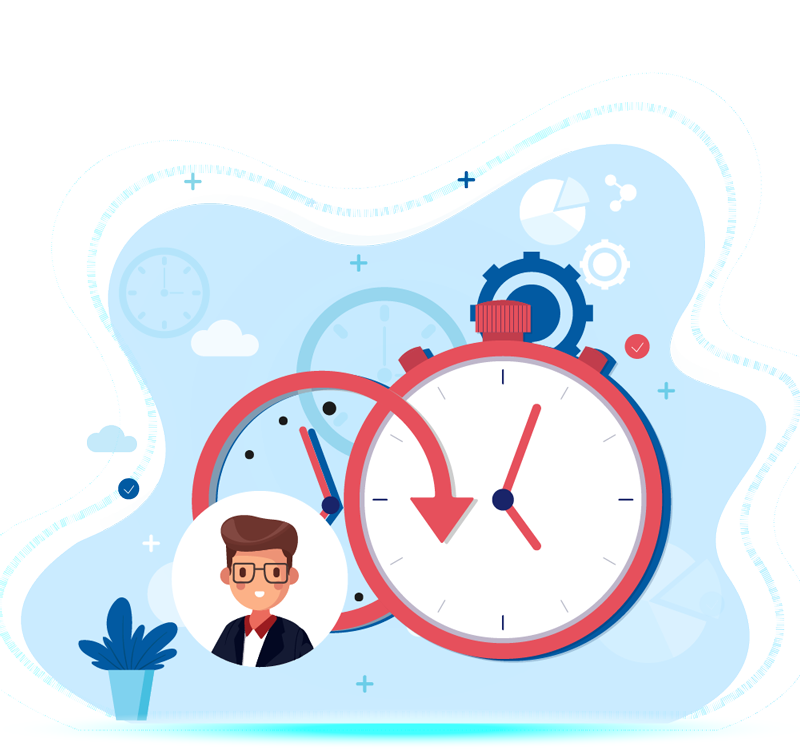

Supplement: Supplemental Information 1 [file peerj-cs-07-678-s001.zip › TIME/TIME/Assets.xcassets/i_nectec_checkin_logo.imageset/i_nectec_checkin_logo.png]

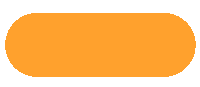

Supplement: Supplemental Information 1 [file peerj-cs-07-678-s001.zip › TIME/TIME/Assets.xcassets/b_main_btn_normal.imageset/b_main_btn_normal.png]

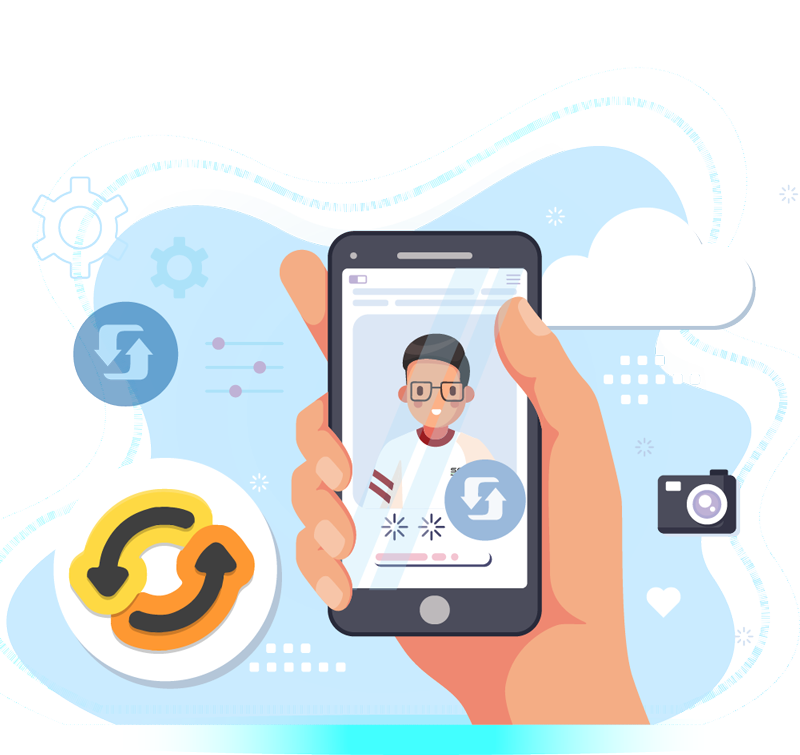

Supplement: Supplemental Information 1 [file peerj-cs-07-678-s001.zip › TIME/TIME/Assets.xcassets/i_nectec_update_logo.imageset/i_nectec_update_logo.png]

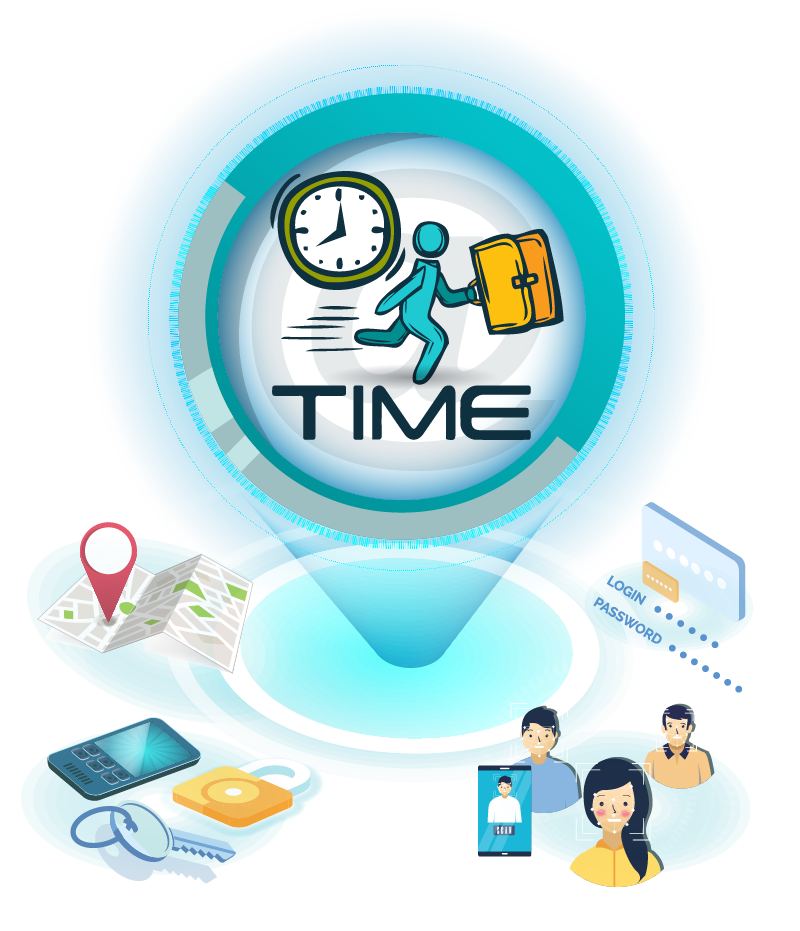

Supplement: Supplemental Information 1 [file peerj-cs-07-678-s001.zip › TIME/TIME/Assets.xcassets/i_nbtc_main_logo1.imageset/i_nbtc_main_logo1.png]

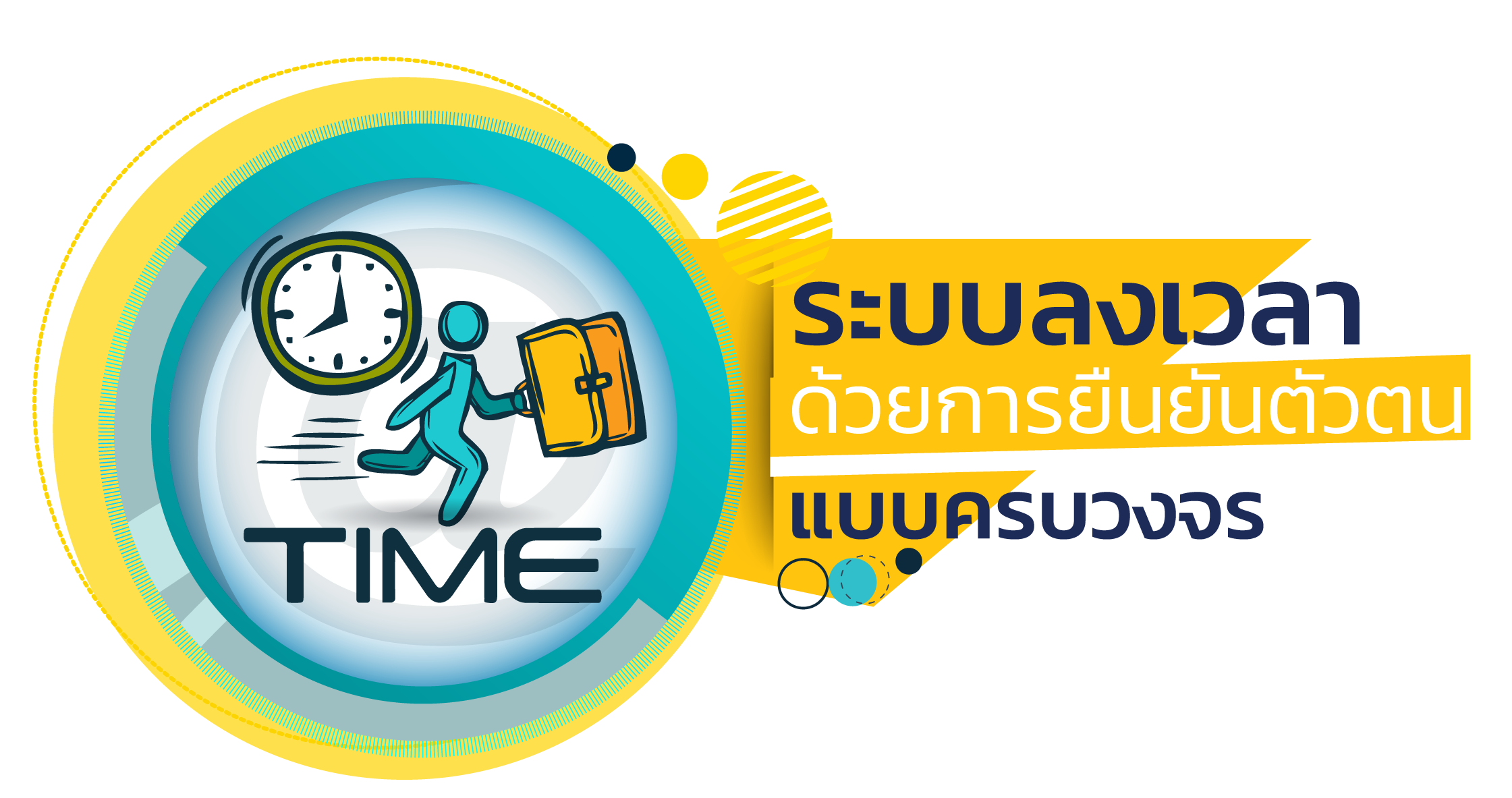

Supplement: Supplemental Information 1 [file peerj-cs-07-678-s001.zip › TIME/TIME/Assets.xcassets/i_papd_logo.imageset/i_papd_logo.png]

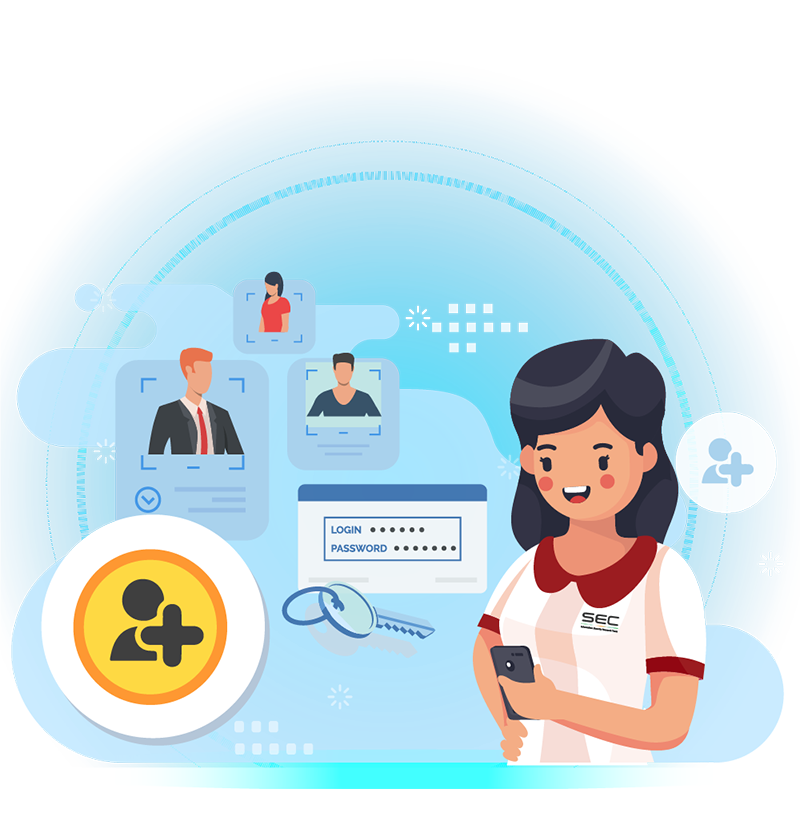

Supplement: Supplemental Information 1 [file peerj-cs-07-678-s001.zip › TIME/TIME/Assets.xcassets/i_nectec_main_logo2.imageset/i_nectec_main_logo2.png]

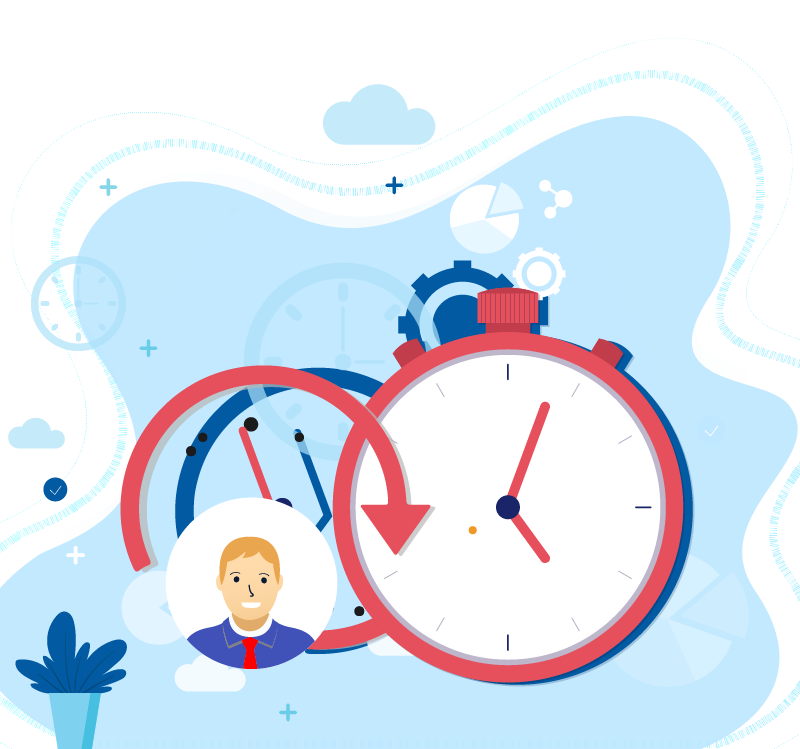

Supplement: Supplemental Information 1 [file peerj-cs-07-678-s001.zip › TIME/TIME/Assets.xcassets/i_nbtc_checkin_logo.imageset/i_nbtc_checkin_logo.png]

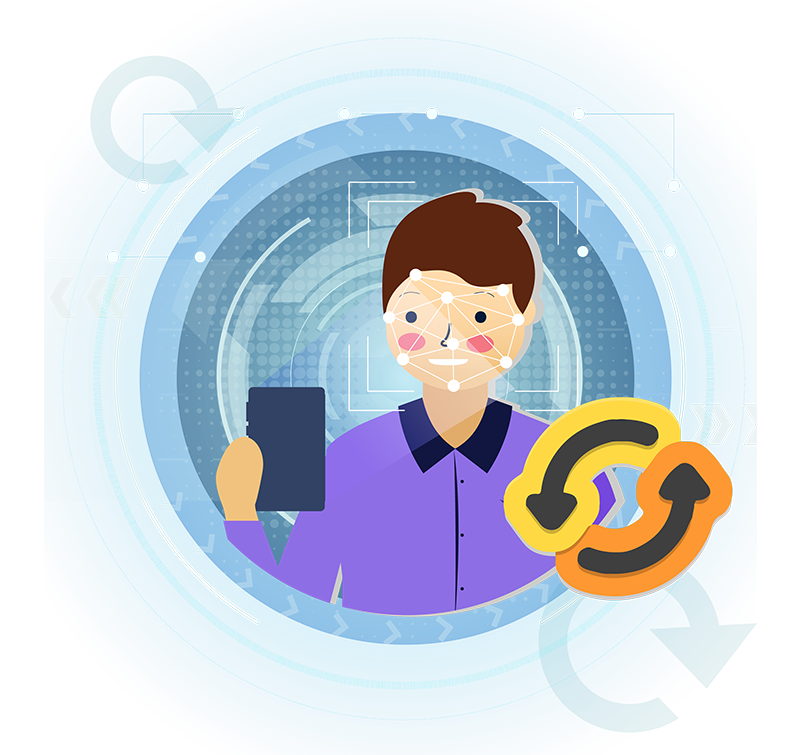

Supplement: Supplemental Information 1 [file peerj-cs-07-678-s001.zip › TIME/TIME/Assets.xcassets/i_nbtc_update_logo.imageset/i_nbtc_update_logo.png]
