# Supplementary material for: Usable comprehensive-factor authentication for a secure time attendance system
Source: PeerJ Comput Sci. 2021 Aug 16;7:e678. doi: 10.7717/peerj-cs.678 (PMC8384039; doi:10.7717/peerj-cs.678)
Supplement: Supplemental Information 2 [file peerj-cs-07-678-s002.pdf]

Raw Data and Results in our Experiment

We have tested up to 100 employees on all four factors. Employee's IDs (ID 001-100) presented are pseudonymized. The results focuses on accuracy in different perspectives. The raw data are presented in four tables/factors.

1. Results on Something you know

The results show an accuracy when employees log in through our system. The accuracy means whether the results of their attempts to login in our system is similar to the results of their attempts to login to the organization's existing Intranet (LDAP/Active Directory). They are asked to make an intention for successful login or login failure (due to invalid password). This is to confirm that in this factor, negative error and positive error will not occur.

Employee ID	Intend to succeed in logging	Intend to fail in logging	Overall Accuracy
001	Accurate (Successful)	Accurate (Access denied)	Accurate
002	Accurate (Successful)	Accurate (Access denied)	Accurate
003	Accurate (Successful)	Accurate (Access denied)	Accurate
004	Accurate (Successful)	Accurate (Access denied)	Accurate
005	Accurate (Successful)	Accurate (Access denied)	Accurate
006	Accurate (Successful)	Accurate (Access denied)	Accurate
007	Accurate (Successful)	Accurate (Access denied)	Accurate
008	Accurate (Successful)	Accurate (Access denied)	Accurate
009	Accurate (Successful)	Accurate (Access denied)	Accurate
010	Accurate (Successful)	Accurate (Access denied)	Accurate
011	Accurate (Successful)	Accurate (Access denied)	Accurate
012	Accurate (Successful)	Accurate (Access denied)	Accurate
013	Accurate (Successful)	Accurate (Access denied)	Accurate
014	Accurate (Successful)	Accurate (Access denied)	Accurate
015	Accurate (Successful)	Accurate (Access denied)	Accurate
016	Accurate (Successful)	Accurate (Access denied)	Accurate
017	Accurate (Successful)	Accurate (Access denied)	Accurate
018	Accurate (Successful)	Accurate (Access denied)	Accurate
019	Accurate (Successful)	Accurate (Access denied)	Accurate
020	Accurate (Successful)	Accurate (Access denied)	Accurate
021	Accurate (Successful)	Accurate (Access denied)	Accurate
022	Accurate (Successful)	Accurate (Access denied)	Accurate
023	Accurate (Successful)	Accurate (Access denied)	Accurate
024	Accurate (Successful)	Accurate (Access denied)	Accurate
025	Accurate (Successful)	Accurate (Access denied)	Accurate
026	Accurate (Successful)	Accurate (Access denied)	Accurate
027	Accurate (Successful)	Accurate (Access denied)	Accurate
028	Accurate (Successful)	Accurate (Access denied)	Accurate
029	Accurate (Successful)	Accurate (Access denied)	Accurate
030	Accurate (Successful)	Accurate (Access denied)	Accurate
031	Accurate (Successful)	Accurate (Access denied)	Accurate
032	Accurate (Successful)	Accurate (Access denied)	Accurate
033	Accurate (Successful)	Accurate (Access denied)	Accurate
034	Accurate (Successful)	Accurate (Access denied)	Accurate
035	Accurate (Successful)	Accurate (Access denied)	Accurate
036	Accurate (Successful)	Accurate (Access denied)	Accurate
037	Accurate (Successful)	Accurate (Access denied)	Accurate
038	Accurate (Successful)	Accurate (Access denied)	Accurate
039	Accurate (Successful)	Accurate (Access denied)	Accurate
040	Accurate (Successful)	Accurate (Access denied)	Accurate
041	Accurate (Successful)	Accurate (Access denied)	Accurate
042	Accurate (Successful)	Accurate (Access denied)	Accurate

043	Accurate (Successful)	Accurate (Access denied)	Accurate
044	Accurate (Successful)	Accurate (Access denied)	Accurate
045	Accurate (Successful)	Accurate (Access denied)	Accurate
046	Accurate (Successful)	Accurate (Access denied)	Accurate
047	Accurate (Successful)	Accurate (Access denied)	Accurate
048	Accurate (Successful)	Accurate (Access denied)	Accurate
049	Accurate (Successful)	Accurate (Access denied)	Accurate
050	Accurate (Successful)	Accurate (Access denied)	Accurate
051	Accurate (Successful)	Accurate (Access denied)	Accurate
052	Accurate (Successful)	Accurate (Access denied)	Accurate
053	Accurate (Successful)	Accurate (Access denied)	Accurate
054	Accurate (Successful)	Accurate (Access denied)	Accurate
055	Accurate (Successful)	Accurate (Access denied)	Accurate
056	Accurate (Successful)	Accurate (Access denied)	Accurate
057	Accurate (Successful)	Accurate (Access denied)	Accurate
058	Accurate (Successful)	Accurate (Access denied)	Accurate
059	Accurate (Successful)	Accurate (Access denied)	Accurate
060	Accurate (Successful)	Accurate (Access denied)	Accurate
061	Accurate (Successful)	Accurate (Access denied)	Accurate
062	Accurate (Successful)	Accurate (Access denied)	Accurate
063	Accurate (Successful)	Accurate (Access denied)	Accurate
064	Accurate (Successful)	Accurate (Access denied)	Accurate
065	Accurate (Successful)	Accurate (Access denied)	Accurate
066	Accurate (Successful)	Accurate (Access denied)	Accurate
067	Accurate (Successful)	Accurate (Access denied)	Accurate
068	Accurate (Successful)	Accurate (Access denied)	Accurate
069	Accurate (Successful)	Accurate (Access denied)	Accurate
070	Accurate (Successful)	Accurate (Access denied)	Accurate
071	Accurate (Successful)	Accurate (Access denied)	Accurate
072	Accurate (Successful)	Accurate (Access denied)	Accurate
073	Accurate (Successful)	Accurate (Access denied)	Accurate
074	Accurate (Successful)	Accurate (Access denied)	Accurate
075	Accurate (Successful)	Accurate (Access denied)	Accurate
076	Accurate (Successful)	Accurate (Access denied)	Accurate
077	Accurate (Successful)	Accurate (Access denied)	Accurate
078	Accurate (Successful)	Accurate (Access denied)	Accurate
079	Accurate (Successful)	Accurate (Access denied)	Accurate
080	Accurate (Successful)	Accurate (Access denied)	Accurate
081	Accurate (Successful)	Accurate (Access denied)	Accurate
082	Accurate (Successful)	Accurate (Access denied)	Accurate
083	Accurate (Successful)	Accurate (Access denied)	Accurate
084	Accurate (Successful)	Accurate (Access denied)	Accurate
085	Accurate (Successful)	Accurate (Access denied)	Accurate
086	Accurate (Successful)	Accurate (Access denied)	Accurate
087	Accurate (Successful)	Accurate (Access denied)	Accurate
088	Accurate (Successful)	Accurate (Access denied)	Accurate
089	Accurate (Successful)	Accurate (Access denied)	Accurate
090	Accurate (Successful)	Accurate (Access denied)	Accurate
091	Accurate (Successful)	Accurate (Access denied)	Accurate
092	Accurate (Successful)	Accurate (Access denied)	Accurate
093	Accurate (Successful)	Accurate (Access denied)	Accurate
094	Accurate (Successful)	Accurate (Access denied)	Accurate
095	Accurate (Successful)	Accurate (Access denied)	Accurate
096	Accurate (Successful)	Accurate (Access denied)	Accurate
097	Accurate (Successful)	Accurate (Access denied)	Accurate
098	Accurate (Successful)	Accurate (Access denied)	Accurate
099	Accurate (Successful)	Accurate (Access denied)	Accurate
100	Accurate (Successful)	Accurate (Access denied)	Accurate

2. Results on Something you have

The results show an accuracy of the results when an employee uses their registered own device and when he/she uses another device.

Employee ID	Use registered own device	Use another unregistered device	Overall Accuracy
001	Accurate (Successful)	Accurate (Access denied)	Accurate
002	Accurate (Successful)	Accurate (Access denied)	Accurate
003	Accurate (Successful)	Accurate (Access denied)	Accurate
004	Accurate (Successful)	Accurate (Access denied)	Accurate
005	Accurate (Successful)	Accurate (Access denied)	Accurate
006	Accurate (Successful)	Accurate (Access denied)	Accurate
007	Accurate (Successful)	Accurate (Access denied)	Accurate
008	Accurate (Successful)	Accurate (Access denied)	Accurate
009	Accurate (Successful)	Accurate (Access denied)	Accurate
010	Accurate (Successful)	Accurate (Access denied)	Accurate
011	Accurate (Successful)	Accurate (Access denied)	Accurate
012	Accurate (Successful)	Accurate (Access denied)	Accurate
013	Accurate (Successful)	Accurate (Access denied)	Accurate
014	Accurate (Successful)	Accurate (Access denied)	Accurate
015	Accurate (Successful)	Accurate (Access denied)	Accurate
016	Accurate (Successful)	Accurate (Access denied)	Accurate
017	Accurate (Successful)	Accurate (Access denied)	Accurate
018	Accurate (Successful)	Accurate (Access denied)	Accurate
019	Accurate (Successful)	Accurate (Access denied)	Accurate
020	Accurate (Successful)	Accurate (Access denied)	Accurate
021	Accurate (Successful)	Accurate (Access denied)	Accurate
022	Accurate (Successful)	Accurate (Access denied)	Accurate
023	Accurate (Successful)	Accurate (Access denied)	Accurate
024	Accurate (Successful)	Accurate (Access denied)	Accurate
025	Accurate (Successful)	Accurate (Access denied)	Accurate
026	Accurate (Successful)	Accurate (Access denied)	Accurate
027	Accurate (Successful)	Accurate (Access denied)	Accurate
028	Accurate (Successful)	Accurate (Access denied)	Accurate
029	Accurate (Successful)	Accurate (Access denied)	Accurate
030	Accurate (Successful)	Accurate (Access denied)	Accurate
031	Accurate (Successful)	Accurate (Access denied)	Accurate
032	Accurate (Successful)	Accurate (Access denied)	Accurate
033	Accurate (Successful)	Accurate (Access denied)	Accurate
034	Accurate (Successful)	Accurate (Access denied)	Accurate
035	Accurate (Successful)	Accurate (Access denied)	Accurate
036	Accurate (Successful)	Accurate (Access denied)	Accurate
037	Accurate (Successful)	Accurate (Access denied)	Accurate
038	Accurate (Successful)	Accurate (Access denied)	Accurate
039	Accurate (Successful)	Accurate (Access denied)	Accurate
040	Accurate (Successful)	Accurate (Access denied)	Accurate
041	Accurate (Successful)	Accurate (Access denied)	Accurate
042	Accurate (Successful)	Accurate (Access denied)	Accurate
043	Accurate (Successful)	Accurate (Access denied)	Accurate
044	Accurate (Successful)	Accurate (Access denied)	Accurate
045	Accurate (Successful)	Accurate (Access denied)	Accurate
046	Accurate (Successful)	Accurate (Access denied)	Accurate
047	Accurate (Successful)	Accurate (Access denied)	Accurate
048	Accurate (Successful)	Accurate (Access denied)	Accurate
049	Accurate (Successful)	Accurate (Access denied)	Accurate
050	Accurate (Successful)	Accurate (Access denied)	Accurate
051	Accurate (Successful)	Accurate (Access denied)	Accurate
052	Accurate (Successful)	Accurate (Access denied)	Accurate
053	Accurate (Successful)	Accurate (Access denied)	Accurate

054	Accurate (Successful)	Accurate (Access denied)	Accurate
055	Accurate (Successful)	Accurate (Access denied)	Accurate
056	Accurate (Successful)	Accurate (Access denied)	Accurate
057	Accurate (Successful)	Accurate (Access denied)	Accurate
058	Accurate (Successful)	Accurate (Access denied)	Accurate
059	Accurate (Successful)	Accurate (Access denied)	Accurate
060	Accurate (Successful)	Accurate (Access denied)	Accurate
061	Accurate (Successful)	Accurate (Access denied)	Accurate
062	Accurate (Successful)	Accurate (Access denied)	Accurate
063	Accurate (Successful)	Accurate (Access denied)	Accurate
064	Accurate (Successful)	Accurate (Access denied)	Accurate
065	Accurate (Successful)	Accurate (Access denied)	Accurate
066	Accurate (Successful)	Accurate (Access denied)	Accurate
067	Accurate (Successful)	Accurate (Access denied)	Accurate
068	Accurate (Successful)	Accurate (Access denied)	Accurate
069	Accurate (Successful)	Accurate (Access denied)	Accurate
070	Accurate (Successful)	Accurate (Access denied)	Accurate
071	Accurate (Successful)	Accurate (Access denied)	Accurate
072	Accurate (Successful)	Accurate (Access denied)	Accurate
073	Accurate (Successful)	Accurate (Access denied)	Accurate
074	Accurate (Successful)	Accurate (Access denied)	Accurate
075	Accurate (Successful)	Accurate (Access denied)	Accurate
076	Accurate (Successful)	Accurate (Access denied)	Accurate
077	Accurate (Successful)	Accurate (Access denied)	Accurate
078	Accurate (Successful)	Accurate (Access denied)	Accurate
079	Accurate (Successful)	Accurate (Access denied)	Accurate
080	Accurate (Successful)	Accurate (Access denied)	Accurate
081	Accurate (Successful)	Accurate (Access denied)	Accurate
082	Accurate (Successful)	Accurate (Access denied)	Accurate
083	Accurate (Successful)	Accurate (Access denied)	Accurate
084	Accurate (Successful)	Accurate (Access denied)	Accurate
085	Accurate (Successful)	Accurate (Access denied)	Accurate
086	Accurate (Successful)	Accurate (Access denied)	Accurate
087	Accurate (Successful)	Accurate (Access denied)	Accurate
088	Accurate (Successful)	Accurate (Access denied)	Accurate
089	Accurate (Successful)	Accurate (Access denied)	Accurate
090	Accurate (Successful)	Accurate (Access denied)	Accurate
091	Accurate (Successful)	Accurate (Access denied)	Accurate
092	Accurate (Successful)	Accurate (Access denied)	Accurate
093	Accurate (Successful)	Accurate (Access denied)	Accurate
094	Accurate (Successful)	Accurate (Access denied)	Accurate
095	Accurate (Successful)	Accurate (Access denied)	Accurate
096	Accurate (Successful)	Accurate (Access denied)	Accurate
097	Accurate (Successful)	Accurate (Access denied)	Accurate
098	Accurate (Successful)	Accurate (Access denied)	Accurate
099	Accurate (Successful)	Accurate (Access denied)	Accurate
100	Accurate (Successful)	Accurate (Access denied)	Accurate

While it is assumable that the MAC can be spoofed, the hacker can get an initial access only in case he/she know the target MAC (registered device). Also, he/she needs to know the target's password. If the hacker fails to obtain one of them data, his/her access will be denied immediately. The results below shows an attempt to access to the system by using a MAC-spoofing device, but (a) the spoofing MAC is registered and (b) the spoofing MAC is not registered, and the hacker does not know the target's password.

Employee ID who is spoofed	Do not know the MAC's target	Know the MAC's target	Overall Accuracy
Not identified #1	Accurate (Access denied)	N/A	Accurate (Access denied)
Not identified #2	Accurate (Access denied)	N/A	Accurate (Access denied)
Not identified #3	Accurate (Access denied)	N/A	Accurate (Access denied)
Not identified #4	Accurate (Access denied)	N/A	Accurate (Access denied)
Not identified #5	Accurate (Access denied)	N/A	Accurate (Access denied)
Not identified #6	Accurate (Access denied)	N/A	Accurate (Access denied)
Not identified #7	Accurate (Access denied)	N/A	Accurate (Access denied)
Not identified #8	Accurate (Access denied)	N/A	Accurate (Access denied)
Not identified #9	Accurate (Access denied)	N/A	Accurate (Access denied)
Not identified #10	Accurate (Access denied)	N/A	Accurate (Access denied)
Not identified #11	Accurate (Access denied)	N/A	Accurate (Access denied)
Not identified #12	Accurate (Access denied)	N/A	Accurate (Access denied)
Not identified #13	Accurate (Access denied)	N/A	Accurate (Access denied)
Not identified #14	Accurate (Access denied)	N/A	Accurate (Access denied)
Not identified #15	Accurate (Access denied)	N/A	Accurate (Access denied)
Not identified #16	Accurate (Access denied)	N/A	Accurate (Access denied)
Not identified #17	Accurate (Access denied)	N/A	Accurate (Access denied)
Not identified #18	Accurate (Access denied)	N/A	Accurate (Access denied)
Not identified #19	Accurate (Access denied)	N/A	Accurate (Access denied)
Not identified #20	Accurate (Access denied)	N/A	Accurate (Access denied)
Not identified #21	Accurate (Access denied)	N/A	Accurate (Access denied)
Not identified #22	Accurate (Access denied)	N/A	Accurate (Access denied)
Not identified #23	Accurate (Access denied)	N/A	Accurate (Access denied)
Not identified #24	Accurate (Access denied)	N/A	Accurate (Access denied)
Not identified #25	Accurate (Access denied)	N/A	Accurate (Access denied)
Not identified #26	Accurate (Access denied)	N/A	Accurate (Access denied)
Not identified #27	Accurate (Access denied)	N/A	Accurate (Access denied)
Not identified #28	Accurate (Access denied)	N/A	Accurate (Access denied)
Not identified #29	Accurate (Access denied)	N/A	Accurate (Access denied)
Not identified #30	Accurate (Access denied)	N/A	Accurate (Access denied)
Not identified #31	Accurate (Access denied)	N/A	Accurate (Access denied)
Not identified #32	Accurate (Access denied)	N/A	Accurate (Access denied)
Not identified #33	Accurate (Access denied)	N/A	Accurate (Access denied)
Not identified #34	Accurate (Access denied)	N/A	Accurate (Access denied)
Not identified #35	Accurate (Access denied)	N/A	Accurate (Access denied)
Not identified #36	Accurate (Access denied)	N/A	Accurate (Access denied)
Not identified #37	Accurate (Access denied)	N/A	Accurate (Access denied)
Not identified #38	Accurate (Access denied)	N/A	Accurate (Access denied)
Not identified #39	Accurate (Access denied)	N/A	Accurate (Access denied)
Not identified #40	Accurate (Access denied)	N/A	Accurate (Access denied)
Not identified #41	Accurate (Access denied)	N/A	Accurate (Access denied)
Not identified #42	Accurate (Access denied)	N/A	Accurate (Access denied)
Not identified #43	Accurate (Access denied)	N/A	Accurate (Access denied)
Not identified #44	Accurate (Access denied)	N/A	Accurate (Access denied)
Not identified #45	Accurate (Access denied)	N/A	Accurate (Access denied)
Not identified #46	Accurate (Access denied)	N/A	Accurate (Access denied)
Not identified #47	Accurate (Access denied)	N/A	Accurate (Access denied)
Not identified #48	Accurate (Access denied)	N/A	Accurate (Access denied)
Not identified #49	Accurate (Access denied)	N/A	Accurate (Access denied)
Not identified #50	Accurate (Access denied)	N/A	Accurate (Access denied)
001	N/A	Accurate (Initial access allowed)	Accurate (Access denied due to invalid password)
002	N/A	Accurate (Initial access allowed)	Accurate (Access denied due to invalid password)
003	N/A	Accurate (Initial access allowed)	Accurate (Access denied due to invalid password)

004	N/A	Accurate (Initial access allowed)	Accurate (Access denied due to invalid password)
005	N/A	Accurate (Initial access allowed)	Accurate (Access denied due to invalid password)
006	N/A	Accurate (Initial access allowed)	Accurate (Access denied due to invalid password)
007	N/A	Accurate (Initial access allowed)	Accurate (Access denied due to invalid password)
008	N/A	Accurate (Initial access allowed)	Accurate (Access denied due to invalid password)
009	N/A	Accurate (Initial access allowed)	Accurate (Access denied due to invalid password)
010	N/A	Accurate (Initial access allowed)	Accurate (Access denied due to invalid password)
011	N/A	Accurate (Initial access allowed)	Accurate (Access denied due to invalid password)
012	N/A	Accurate (Initial access allowed)	Accurate (Access denied due to invalid password)
013	N/A	Accurate (Initial access allowed)	Accurate (Access denied due to invalid password)
014	N/A	Accurate (Initial access allowed)	Accurate (Access denied due to invalid password)
015	N/A	Accurate (Initial access allowed)	Accurate (Access denied due to invalid password)
016	N/A	Accurate (Initial access allowed)	Accurate (Access denied due to invalid password)
017	N/A	Accurate (Initial access allowed)	Accurate (Access denied due to invalid password)
018	N/A	Accurate (Initial access allowed)	Accurate (Access denied due to invalid password)
019	N/A	Accurate (Initial access allowed)	Accurate (Access denied due to invalid password)
020	N/A	Accurate (Initial access allowed)	Accurate (Access denied due to invalid password)
021	N/A	Accurate (Initial access allowed)	Accurate (Access denied due to invalid password)
022	N/A	Accurate (Initial access allowed)	Accurate (Access denied due to invalid password)
023	N/A	Accurate (Initial access allowed)	Accurate (Access denied due to invalid password)
024	N/A	Accurate (Initial access allowed)	Accurate (Access denied due to invalid password)
025	N/A	Accurate (Initial access allowed)	Accurate (Access denied due to invalid password)
026	N/A	Accurate (Initial access allowed)	Accurate (Access denied due to invalid password)
027	N/A	Accurate (Initial access allowed)	Accurate (Access denied due to invalid password)
028	N/A	Accurate (Initial access allowed)	Accurate (Access denied due to invalid password)
029	N/A	Accurate (Initial access allowed)	Accurate (Access denied due to invalid password)
030	N/A	Accurate (Initial access allowed)	Accurate (Access denied due to invalid password)
031	N/A	Accurate (Initial access allowed)	Accurate (Access denied due to invalid password)
032	N/A	Accurate (Initial access allowed)	Accurate (Access denied due to invalid password)
033	N/A	Accurate (Initial access allowed)	Accurate (Access denied due to invalid password)

034	N/A	Accurate (Initial access allowed)	Accurate (Access denied due to invalid password)
035	N/A	Accurate (Initial access allowed)	Accurate (Access denied due to invalid password)
036	N/A	Accurate (Initial access allowed)	Accurate (Access denied due to invalid password)
037	N/A	Accurate (Initial access allowed)	Accurate (Access denied due to invalid password)
038	N/A	Accurate (Initial access allowed)	Accurate (Access denied due to invalid password)
039	N/A	Accurate (Initial access allowed)	Accurate (Access denied due to invalid password)
040	N/A	Accurate (Initial access allowed)	Accurate (Access denied due to invalid password)
041	N/A	Accurate (Initial access allowed)	Accurate (Access denied due to invalid password)
042	N/A	Accurate (Initial access allowed)	Accurate (Access denied due to invalid password)
043	N/A	Accurate (Initial access allowed)	Accurate (Access denied due to invalid password)
044	N/A	Accurate (Initial access allowed)	Accurate (Access denied due to invalid password)
045	N/A	Accurate (Initial access allowed)	Accurate (Access denied due to invalid password)
046	N/A	Accurate (Initial access allowed)	Accurate (Access denied due to invalid password)
047	N/A	Accurate (Initial access allowed)	Accurate (Access denied due to invalid password)
048	N/A	Accurate (Initial access allowed)	Accurate (Access denied due to invalid password)
049	N/A	Accurate (Initial access allowed)	Accurate (Access denied due to invalid password)
050	N/A	Accurate (Initial access allowed)	Accurate (Access denied due to invalid password)

3. Results on Something you are

The results show an accuracy of the results when an employee scan his/her face after passing the two previous factors. Employee ID 89 and 98 are identical twins that our system is unable to differentiate. Therefore, the overall accuracy is 98%, while the negative error rate is 2%. It is assumable that these twins do not share password and device to each other, thus they finally do not get an access or cannot check-in on behalf successfully.

Employee ID	Use own face	Use another face	Overall Accuracy
001	Accurate (Successful)	Accurate (Access denied)	Accurate
002	Accurate (Successful)	Accurate (Access denied)	Accurate
003	Accurate (Successful)	Accurate (Access denied)	Accurate
004	Accurate (Successful)	Accurate (Access denied)	Accurate
005	Accurate (Successful)	Accurate (Access denied)	Accurate
006	Accurate (Successful)	Accurate (Access denied)	Accurate
007	Accurate (Successful)	Accurate (Access denied)	Accurate
008	Accurate (Successful)	Accurate (Access denied)	Accurate
009	Accurate (Successful)	Accurate (Access denied)	Accurate
010	Accurate (Successful)	Accurate (Access denied)	Accurate
011	Accurate (Successful)	Accurate (Access denied)	Accurate
012	Accurate (Successful)	Accurate (Access denied)	Accurate
013	Accurate (Successful)	Accurate (Access denied)	Accurate

014	Accurate (Successful)	Accurate (Access denied)	Accurate
015	Accurate (Successful)	Accurate (Access denied)	Accurate
016	Accurate (Successful)	Accurate (Access denied)	Accurate
017	Accurate (Successful)	Accurate (Access denied)	Accurate
018	Accurate (Successful)	Accurate (Access denied)	Accurate
019	Accurate (Successful)	Accurate (Access denied)	Accurate
020	Accurate (Successful)	Accurate (Access denied)	Accurate
021	Accurate (Successful)	Accurate (Access denied)	Accurate
022	Accurate (Successful)	Accurate (Access denied)	Accurate
023	Accurate (Successful)	Accurate (Access denied)	Accurate
024	Accurate (Successful)	Accurate (Access denied)	Accurate
025	Accurate (Successful)	Accurate (Access denied)	Accurate
026	Accurate (Successful)	Accurate (Access denied)	Accurate
027	Accurate (Successful)	Accurate (Access denied)	Accurate
028	Accurate (Successful)	Accurate (Access denied)	Accurate
029	Accurate (Successful)	Accurate (Access denied)	Accurate
030	Accurate (Successful)	Accurate (Access denied)	Accurate
031	Accurate (Successful)	Accurate (Access denied)	Accurate
032	Accurate (Successful)	Accurate (Access denied)	Accurate
033	Accurate (Successful)	Accurate (Access denied)	Accurate
034	Accurate (Successful)	Accurate (Access denied)	Accurate
035	Accurate (Successful)	Accurate (Access denied)	Accurate
036	Accurate (Successful)	Accurate (Access denied)	Accurate
037	Accurate (Successful)	Accurate (Access denied)	Accurate
038	Accurate (Successful)	Accurate (Access denied)	Accurate
039	Accurate (Successful)	Accurate (Access denied)	Accurate
040	Accurate (Successful)	Accurate (Access denied)	Accurate
041	Accurate (Successful)	Accurate (Access denied)	Accurate
042	Accurate (Successful)	Accurate (Access denied)	Accurate
043	Accurate (Successful)	Accurate (Access denied)	Accurate
044	Accurate (Successful)	Accurate (Access denied)	Accurate
045	Accurate (Successful)	Accurate (Access denied)	Accurate
046	Accurate (Successful)	Accurate (Access denied)	Accurate
047	Accurate (Successful)	Accurate (Access denied)	Accurate
048	Accurate (Successful)	Accurate (Access denied)	Accurate
049	Accurate (Successful)	Accurate (Access denied)	Accurate
050	Accurate (Successful)	Accurate (Access denied)	Accurate
051	Accurate (Successful)	Accurate (Access denied)	Accurate
052	Accurate (Successful)	Accurate (Access denied)	Accurate
053	Accurate (Successful)	Accurate (Access denied)	Accurate
054	Accurate (Successful)	Accurate (Access denied)	Accurate
055	Accurate (Successful)	Accurate (Access denied)	Accurate
056	Accurate (Successful)	Accurate (Access denied)	Accurate
057	Accurate (Successful)	Accurate (Access denied)	Accurate
058	Accurate (Successful)	Accurate (Access denied)	Accurate
059	Accurate (Successful)	Accurate (Access denied)	Accurate
060	Accurate (Successful)	Accurate (Access denied)	Accurate
061	Accurate (Successful)	Accurate (Access denied)	Accurate
062	Accurate (Successful)	Accurate (Access denied)	Accurate
063	Accurate (Successful)	Accurate (Access denied)	Accurate
064	Accurate (Successful)	Accurate (Access denied)	Accurate
065	Accurate (Successful)	Accurate (Access denied)	Accurate
066	Accurate (Successful)	Accurate (Access denied)	Accurate
067	Accurate (Successful)	Accurate (Access denied)	Accurate
068	Accurate (Successful)	Accurate (Access denied)	Accurate
069	Accurate (Successful)	Accurate (Access denied)	Accurate
070	Accurate (Successful)	Accurate (Access denied)	Accurate
071	Accurate (Successful)	Accurate (Access denied)	Accurate
072	Accurate (Successful)	Accurate (Access denied)	Accurate

073	Accurate (Successful)	Accurate (Access denied)	Accurate
074	Accurate (Successful)	Accurate (Access denied)	Accurate
075	Accurate (Successful)	Accurate (Access denied)	Accurate
076	Accurate (Successful)	Accurate (Access denied)	Accurate
077	Accurate (Successful)	Accurate (Access denied)	Accurate
078	Accurate (Successful)	Accurate (Access denied)	Accurate
079	Accurate (Successful)	Accurate (Access denied)	Accurate
080	Accurate (Successful)	Accurate (Access denied)	Accurate
081	Accurate (Successful)	Accurate (Access denied)	Accurate
082	Accurate (Successful)	Accurate (Access denied)	Accurate
083	Accurate (Successful)	Accurate (Access denied)	Accurate
084	Accurate (Successful)	Accurate (Access denied)	Accurate
085	Accurate (Successful)	Accurate (Access denied)	Accurate
086	Accurate (Successful)	Accurate (Access denied)	Accurate
087	Accurate (Successful)	Accurate (Access denied)	Accurate
088	Accurate (Successful)	Accurate (Access denied)	Accurate
089 (identical twin to 098)	Accurate (Successful)	Inaccurate (Access allowed) – using the face of ID 098	Inaccurate
090	Accurate (Successful)	Accurate (Access denied)	Accurate
091	Accurate (Successful)	Accurate (Access denied)	Accurate
092	Accurate (Successful)	Accurate (Access denied)	Accurate
093	Accurate (Successful)	Accurate (Access denied)	Accurate
094	Accurate (Successful)	Accurate (Access denied)	Accurate
095	Accurate (Successful)	Accurate (Access denied)	Accurate
096	Accurate (Successful)	Accurate (Access denied)	Accurate
097	Accurate (Successful)	Accurate (Access denied)	Accurate
098 (identical twin to 089)	Accurate (Successful)	Inaccurate (Access allowed) – using the face of ID 089	Inaccurate
099	Accurate (Successful)	Accurate (Access denied)	Accurate
100	Accurate (Successful)	Accurate (Access denied)	Accurate

4. Results on Somewhere you are

The results show the results of when an employee accesses within a designated area, outside corporate WiFi and via a corporate VPN.

Employee ID	Within a designated area	Outside corporate WiFi	Via a corporate VPN	Overall accuracy
001	Accurate (Successful)	Accurate (Access denied)	Accurate (Access denied)	Accurate
002	Accurate (Successful)	Accurate (Access denied)	Accurate (Access denied)	Accurate
003	Accurate (Successful)	Accurate (Access denied)	Accurate (Access denied)	Accurate
004	Accurate (Successful)	Accurate (Access denied)	Accurate (Access denied)	Accurate
005	Accurate (Successful)	Accurate (Access denied)	Accurate (Access denied)	Accurate
006	Accurate (Successful)	Accurate (Access denied)	Accurate (Access denied)	Accurate
007	Accurate (Successful)	Accurate (Access denied)	Accurate (Access denied)	Accurate
008	Accurate (Successful)	Accurate (Access denied)	Accurate (Access denied)	Accurate
009	Accurate (Successful)	Accurate (Access denied)	Accurate (Access denied)	Accurate
010	Accurate (Successful)	Accurate (Access denied)	Accurate (Access denied)	Accurate
011	Accurate (Successful)	Accurate (Access denied)	Accurate (Access denied)	Accurate
012	Accurate (Successful)	Accurate (Access denied)	Accurate (Access denied)	Accurate
013	Accurate (Successful)	Accurate (Access denied)	Accurate (Access denied)	Accurate
014	Accurate (Successful)	Accurate (Access denied)	Accurate (Access denied)	Accurate
015	Accurate (Successful)	Accurate (Access denied)	Accurate (Access denied)	Accurate
016	Accurate (Successful)	Accurate (Access denied)	Accurate (Access denied)	Accurate
017	Accurate (Successful)	Accurate (Access denied)	Accurate (Access denied)	Accurate
018	Accurate (Successful)	Accurate (Access denied)	Accurate (Access denied)	Accurate
019	Accurate (Successful)	Accurate (Access denied)	Accurate (Access denied)	Accurate

020	Accurate (Successful)	Accurate (Access denied)	Accurate (Access denied)	Accurate
021	Accurate (Successful)	Accurate (Access denied)	Accurate (Access denied)	Accurate
022	Accurate (Successful)	Accurate (Access denied)	Accurate (Access denied)	Accurate
023	Accurate (Successful)	Accurate (Access denied)	Accurate (Access denied)	Accurate
024	Accurate (Successful)	Accurate (Access denied)	Accurate (Access denied)	Accurate
025	Accurate (Successful)	Accurate (Access denied)	Accurate (Access denied)	Accurate
026	Accurate (Successful)	Accurate (Access denied)	Accurate (Access denied)	Accurate
027	Accurate (Successful)	Accurate (Access denied)	Accurate (Access denied)	Accurate
028	Accurate (Successful)	Accurate (Access denied)	Accurate (Access denied)	Accurate
029	Accurate (Successful)	Accurate (Access denied)	Accurate (Access denied)	Accurate
030	Accurate (Successful)	Accurate (Access denied)	Accurate (Access denied)	Accurate
031	Accurate (Successful)	Accurate (Access denied)	Accurate (Access denied)	Accurate
032	Accurate (Successful)	Accurate (Access denied)	Accurate (Access denied)	Accurate
033	Accurate (Successful)	Accurate (Access denied)	Accurate (Access denied)	Accurate
034	Accurate (Successful)	Accurate (Access denied)	Accurate (Access denied)	Accurate
035	Accurate (Successful)	Accurate (Access denied)	Accurate (Access denied)	Accurate
036	Accurate (Successful)	Accurate (Access denied)	Accurate (Access denied)	Accurate
037	Accurate (Successful)	Accurate (Access denied)	Accurate (Access denied)	Accurate
038	Accurate (Successful)	Accurate (Access denied)	Accurate (Access denied)	Accurate
039	Accurate (Successful)	Accurate (Access denied)	Accurate (Access denied)	Accurate
040	Accurate (Successful)	Accurate (Access denied)	Accurate (Access denied)	Accurate
041	Accurate (Successful)	Accurate (Access denied)	Accurate (Access denied)	Accurate
042	Accurate (Successful)	Accurate (Access denied)	Accurate (Access denied)	Accurate
043	Accurate (Successful)	Accurate (Access denied)	Accurate (Access denied)	Accurate
044	Accurate (Successful)	Accurate (Access denied)	Accurate (Access denied)	Accurate
045	Accurate (Successful)	Accurate (Access denied)	Accurate (Access denied)	Accurate
046	Accurate (Successful)	Accurate (Access denied)	Accurate (Access denied)	Accurate
047	Accurate (Successful)	Accurate (Access denied)	Accurate (Access denied)	Accurate
048	Accurate (Successful)	Accurate (Access denied)	Accurate (Access denied)	Accurate
049	Accurate (Successful)	Accurate (Access denied)	Accurate (Access denied)	Accurate
050	Accurate (Successful)	Accurate (Access denied)	Accurate (Access denied)	Accurate
051	Accurate (Successful)	Accurate (Access denied)	Accurate (Access denied)	Accurate
052	Accurate (Successful)	Accurate (Access denied)	Accurate (Access denied)	Accurate
053	Accurate (Successful)	Accurate (Access denied)	Accurate (Access denied)	Accurate
054	Accurate (Successful)	Accurate (Access denied)	Accurate (Access denied)	Accurate
055	Accurate (Successful)	Accurate (Access denied)	Accurate (Access denied)	Accurate
056	Accurate (Successful)	Accurate (Access denied)	Accurate (Access denied)	Accurate
057	Accurate (Successful)	Accurate (Access denied)	Accurate (Access denied)	Accurate
058	Accurate (Successful)	Accurate (Access denied)	Accurate (Access denied)	Accurate
059	Accurate (Successful)	Accurate (Access denied)	Accurate (Access denied)	Accurate
060	Accurate (Successful)	Accurate (Access denied)	Accurate (Access denied)	Accurate
061	Accurate (Successful)	Accurate (Access denied)	Accurate (Access denied)	Accurate
062	Accurate (Successful)	Accurate (Access denied)	Accurate (Access denied)	Accurate
063	Accurate (Successful)	Accurate (Access denied)	Accurate (Access denied)	Accurate
064	Accurate (Successful)	Accurate (Access denied)	Accurate (Access denied)	Accurate
065	Accurate (Successful)	Accurate (Access denied)	Accurate (Access denied)	Accurate
066	Accurate (Successful)	Accurate (Access denied)	Accurate (Access denied)	Accurate
067	Accurate (Successful)	Accurate (Access denied)	Accurate (Access denied)	Accurate
068	Accurate (Successful)	Accurate (Access denied)	Accurate (Access denied)	Accurate
069	Accurate (Successful)	Accurate (Access denied)	Accurate (Access denied)	Accurate
070	Accurate (Successful)	Accurate (Access denied)	Accurate (Access denied)	Accurate
071	Accurate (Successful)	Accurate (Access denied)	Accurate (Access denied)	Accurate
072	Accurate (Successful)	Accurate (Access denied)	Accurate (Access denied)	Accurate
073	Accurate (Successful)	Accurate (Access denied)	Accurate (Access denied)	Accurate
074	Accurate (Successful)	Accurate (Access denied)	Accurate (Access denied)	Accurate
075	Accurate (Successful)	Accurate (Access denied)	Accurate (Access denied)	Accurate
076	Accurate (Successful)	Accurate (Access denied)	Accurate (Access denied)	Accurate
077	Accurate (Successful)	Accurate (Access denied)	Accurate (Access denied)	Accurate
078	Accurate (Successful)	Accurate (Access denied)	Accurate (Access denied)	Accurate

079	Accurate (Successful)	Accurate (Access denied)	Accurate (Access denied)	Accurate
080	Accurate (Successful)	Accurate (Access denied)	Accurate (Access denied)	Accurate
081	Accurate (Successful)	Accurate (Access denied)	Accurate (Access denied)	Accurate
082	Accurate (Successful)	Accurate (Access denied)	Accurate (Access denied)	Accurate
083	Accurate (Successful)	Accurate (Access denied)	Accurate (Access denied)	Accurate
084	Accurate (Successful)	Accurate (Access denied)	Accurate (Access denied)	Accurate
085	Accurate (Successful)	Accurate (Access denied)	Accurate (Access denied)	Accurate
086	Accurate (Successful)	Accurate (Access denied)	Accurate (Access denied)	Accurate
087	Accurate (Successful)	Accurate (Access denied)	Accurate (Access denied)	Accurate
088	Accurate (Successful)	Accurate (Access denied)	Accurate (Access denied)	Accurate
089	Accurate (Successful)	Accurate (Access denied)	Accurate (Access denied)	Accurate
090	Accurate (Successful)	Accurate (Access denied)	Accurate (Access denied)	Accurate
091	Accurate (Successful)	Accurate (Access denied)	Accurate (Access denied)	Accurate
092	Accurate (Successful)	Accurate (Access denied)	Accurate (Access denied)	Accurate
093	Accurate (Successful)	Accurate (Access denied)	Accurate (Access denied)	Accurate
094	Accurate (Successful)	Accurate (Access denied)	Accurate (Access denied)	Accurate
095	Accurate (Successful)	Accurate (Access denied)	Accurate (Access denied)	Accurate
096	Accurate (Successful)	Accurate (Access denied)	Accurate (Access denied)	Accurate
097	Accurate (Successful)	Accurate (Access denied)	Accurate (Access denied)	Accurate
098	Accurate (Successful)	Accurate (Access denied)	Accurate (Access denied)	Accurate
099	Accurate (Successful)	Accurate (Access denied)	Accurate (Access denied)	Accurate
100	Accurate (Successful)	Accurate (Access denied)	Accurate (Access denied)	Accurate
